# Joint Modeling of Individual Trajectories, Within-Individual Variability, and a Later Outcome: Systolic Blood Pressure Through Childhood and Left Ventricular Mass in Early Adulthood

**DOI:** 10.1093/aje/kwaa224

**Published:** 2020-10-15

**Authors:** Richard M A Parker, George Leckie, Harvey Goldstein, Laura D Howe, Jon Heron, Alun D Hughes, David M Phillippo, Kate Tilling

**Keywords:** ALSPAC, Bayesian analysis, blood pressure, children, joint model, left ventricular hypertrophy, longitudinal studies, young adult

## Abstract

Within-individual variability of repeatedly measured exposures might predict later outcomes (e.g., blood pressure (BP) variability (BPV) is an independent cardiovascular risk factor above and beyond mean BP). Because 2-stage methods, known to introduce bias, are typically used to investigate such associations, we introduce a joint modeling approach, examining associations of mean BP and BPV across childhood with left ventricular mass (indexed to height; LVMI) in early adulthood with data (collected 1990–2011) from the UK Avon Longitudinal Study of Parents and Children cohort. Using multilevel models, we allowed BPV to vary between individuals (a “random effect”) as well as to depend on covariates (allowing for heteroskedasticity). We further distinguished within-clinic variability (“measurement error”) from visit-to-visit BPV. BPV was predicted to be greater at older ages, at higher body weights, and in female participants and was positively correlated with mean BP. BPV had a weak positive association with LVMI (10% increase in within-individual BP variance was predicted to increase LVMI by 0.21%, 95% credible interval: −0.23, 0.69), but this association became negative (−0.78%, 95% credible interval: −2.54, 0.22) once the effect of mean BP on LVMI was adjusted for. This joint modeling approach offers a flexible method of relating repeatedly measured exposures to later outcomes.

## Abbreviations

ALSPAC: Avon Longitudinal Study of Parents and Children
BP: blood pressure
BPV: blood pressure variability
CrI: credible interval
LVMI: left ventricular mass indexed to height
SBP: systolic blood pressure
SD: standard deviation

When exposures vary over time—for example, systolic blood pressure (BP) as measured over the life course—both the overall mean and the change in the exposure can affect later health outcomes (1). However, within-individual variability (i.e., the extent to which an individual’s measurements fluctuates around this trend) might also be a risk factor (2).

Methodological difficulties have meant that within-individual variability in time-varying exposures have seldom been examined, and when they are it is often using 2-stage procedures, known to introduce bias (3). An alternative, 1-stage approach is to use joint multilevel models to relate time-varying exposures (4) to a later outcome (5). These models have recently been extended to also examine within-individual variability (3). Such joint models lead to unbiased parameter estimates and correct standard errors, either when relating the mean and trajectory (6) or the within-individual variability (3), of an exposure to a later outcome.

We illustrate this approach by examining the association of both mean BP and within-individual blood pressure variability (BPV)—more specifically, the estimated individual deviation away from the population mean BP and BPV (adjusted for covariates)—with an established biomarker of target organ damage: left ventricular mass indexed to height (LVMI). While BPV can be measured across various time scales (7), here we focus on the longer term, also called “visit-to-visit.” In adults, visit-to-visit BPV has been found to be an important predictor of subsequent cardiovascular disease over and above mean level (8). Detecting those at risk of cardiovascular disease early in life is important in order to design and administer preventive measures in a timely and targeted manner (9, 10). Mean systolic BP in childhood is positively associated with early signs of heart damage, such as left ventricular hypertrophy (11–13), and there is evidence from a US cohort of children that higher BPV—as estimated via the standard deviation (SD) of the measurements across all visits (conducted every 2–3 years) for each person—is associated with adult hypertension, independent of mean BP levels (14). However, there has been little investigation of the role of BPV on early target-organ damage, despite its potential utility as a predictive factor (15), and only limited analysis of the factors associated with childhood BPV (14, 16).

Our aim was to explore the factors associated with within-individual BPV in a UK cohort of children using multilevel analyses, and to extend these to joint models to investigate the association of both mean BP and BPV with LVMI in early adulthood. Note that, because the responses are both Gaussian, the model could also be referred to a multivariate normal response model, with heteroskedastic error.

## METHODS

### Participants

The participants were from the UK’s Avon Longitudinal Study of Parents and Children (ALSPAC), an ongoing, prospective longitudinal birth cohort study (17–19). Pregnant women resident in Avon, with expected dates of delivery of April 1, 1991, to December 31, 1992, were invited to take part in the study; 14,541 pregnancies, resulting in 13,988 children alive at 1 year of age, were initially enrolled, later bolstered by further eligible cases who had failed to join the study originally, increasing the number of enrolled pregnancies to 15,454, with 14,901 children alive at 1 year of age. The study website contains details of all available data through a fully searchable data dictionary and variable search tool (20).

A total of 5,217 of the participants attended an ALSPAC study clinic at a mean age of 17.7 years, of whom a random subsample of 2,047 (all singletons) had their LVMI (left ventricular mass in grams, indexed to height in meters^2.7^ (g/m^2.7^)) measured via echocardiography (21, 22). Exclusion criteria included pregnancy and congenital heart disease; see below for a comparison of the subsample modeled with the larger sample enrolled in ALSPAC and Boyd et al. (17) for a discussion of attrition in ALSPAC. Echocardiography was performed using an HDI 5000 ultrasound machine equipped with a P4-2 Phased Array ultrasound transducer (Philips Healthcare, Amsterdam, The Netherlands) by one of 2 experienced echocardiographers using a standard examination protocol (22).

A total of 1,988 of these participants had their systolic BP (SBP), and their height, weight, and age recorded on at least 1 prior occasion (research clinic). These clinics’ participants were approximately 7.5, 9.5, 10.5, 11.5, 13, and 15.5 years of age. At each clinic, the participant’s SBP (in mm Hg) was measured at least twice, using a validated electronic monitoring device and a cuff size appropriate for their upper arm circumference, with the participant sitting and at rest with the arm supported. Each of these measurements was available for our analysis from each clinic, apart from the clinic at approximately 10.5 years of age, for which only the mean was available.

### Statistical analysis

The covariates we included for SBP were age (for further details, see below), sex, a term for interaction between age and sex, weight, height, and a number of maternal characteristics, including parity (the number of previous pregnancies resulting in either livebirth or stillbirth), age at delivery, and highest educational qualification. Sex, age, and weight, as measured at the clinic at approximately 17.7 years of age, were included as exposures for LVMI, but height was not, because LVMI is indexed to height. All continuous covariates were centered around their grand mean prior to analysis. Weights, as measured across childhood, and LVMI were log-transformed prior to analysis.

We used a joint model to relate BPV to subsequent log(LVMI). We describe this modeling approach below, building up model complexity using an example that assumes that SBP increases linearly with age across childhood (we later relax this assumption in the ALSPAC model). Equation 1 assumes that each individual has 1 measure at each occasion, but this is relaxed in equation 2 (and in the ALSPAC model).

(1) \begin{align*} {y}_{1 ij}={\beta}_0+{\beta}_1{t}_{ij}+{u}_{0j}+{u}_{1j}{t}_{ij}+{e}_{ij} \quad\quad\quad\quad\quad\quad\quad\quad\quad \ \ \nonumber\\{e}_{ij}\sim N\big(0,{\sigma}_{eij}^2\big), \log \big({\sigma}_{eij}^2\big)={\alpha}_0+{\alpha}_1{t}_{ij}+{u}_{2j}\quad\quad\quad\quad\quad\quad\nonumber\\{y}_{2j}={\gamma}_0+{\gamma}_1{u}_{0j}+{\gamma}_2{u}_{1j}+ {\gamma}_3{u}_{2j}+{u}_{3j}\quad\quad\quad\quad\quad\quad \ \nonumber \quad \ \ \ \\\left(\begin{array}{@{}c@{}}{u}_{0j}\\{}\begin{array}{c}{u}_{1j}\\{}{u}_{2j}\\{}{u}_{3j}\end{array}\end{array}\right) \sim N\left\{\!\left(\begin{array}{@{}c@{}}0\\{}\begin{array}{c}0\\{}0\\{}0\end{array}\end{array}\right)\!,\left(\!\begin{array}{@{}c@{}}{\sigma}_{\mathrm{u}0}^2\kern8.em \\{}\begin{array}{c}{\sigma}_{\mathrm{u}01}\kern1em {\sigma}_{u1}^2\kern5.75em \\{}{\sigma}_{u02\kern0.75em }\kern0.5em {\sigma}_{u12}\kern0.75em {\sigma}_{u2}^2\kern3em \\{}0\kern2.25em 0\kern2.25em 0\kern1.5em {\sigma}_{u3}^2\end{array}\!\!\!\!\!\end{array}\right)\right\} \end{align*}

In equation 1, ${y}_{1 ij}$ is the repeatedly measured exposure (SBP) measured at clinic *i* (*i* = 1, …, ${n}_j$) for individual *j* (*j* = 1, …, J), with the covariate ${t}_{ij}$ indicating the individual’s age at that clinic. In the mean function for BP, there are individual-level random effects for the intercept (${u}_{0j}$; mean BP at $t$ = 0) and also for rate of change (${u}_{1j}$; BP slope), with within-individual (between-clinic) error ${e}_{ij}$. In standard multilevel models, the variance of the within-individual error is assumed to be constant (homoskedastic) across all observations (as ${\sigma}_e^2$). Here it is allowed to depend on age (${t}_{ij}$) and also on an individual-level random effect ${u}_{2j}$ (with a log-link ensuring that the within-individual variance remains positive). Within-individual variability in blood pressure, log(BPV), is therefore allowed to change with age (as estimated here via ${\alpha}_1$). The random effect ${u}_{2j}$ allows each individual to have their own estimate of log(BPV). It is also possible to allow for coefficients of covariates within the within-individual variability function to randomly vary across individual: for example, allowing the coefficient for age (${t}_{ij}$) to randomly vary to investigate whether its association with BPV differs between people (23). The submodel for the longitudinal process is a mixed-effects location scale model (24, 25). The inclusion of the individual-level outcome ${y}_{2j}$, denoting log(LVMI) measured at approximately 17.7 years for individual *j* (*j* = 1, …, J), results in a joint model, with shared random effects: here via the inclusion of mean BP, BP slope, and log(BPV) as exposures in the linear model for the mean of log(LVMI).

The model above has 2 levels, and assumes there is just 1 measurement per person, per clinic. If more than 1 measurement of SBP is taken for each individual at each clinic session, however, then the model can be expanded as follows:

(2) \begin{align*} &{y}_{1 hij}={\beta}_0\!+{\beta}_1{t}_{ij}\!+{u}_{0j}\!+{\!u}_{1j}{t}_{ij}+{e}_{ij}+\!{\varepsilon}_{hij}\quad\quad\quad\quad\quad\quad \nonumber\\ &{e}_{ij}\!\sim\! N\big(0,{\sigma}_{eij}^2\big), \log\! \big({\sigma}_{eij}^2\big)={\alpha}_0+{\alpha}_1{t}_{ij}+{u}_{2j}\quad\quad\quad\quad\quad\nonumber\\ &{\varepsilon}_{hij}\sim N\big(0,{\sigma}_{\varepsilon}^2\big)\ \ \nonumber\\ &{y}_{2j}={\gamma}_0+{\gamma}_1{u}_{0j}+{\gamma}_2{u}_{1j}+{\gamma}_3{u}_{2j}+{u}_{3j}\ \nonumber\\& \left(\begin{array}{@{}c@{}}{u}_{0j}\\{}\begin{array}{c}{u}_{1j}\\{}{u}_{2j}\\{}{u}_{3j}\end{array}\end{array}\right) \sim N\left\{\left(\begin{array}{@{}c@{}}0\\{}\begin{array}{c}0\\{}0\\{}0\end{array}\end{array}\right)\!,\left(\!\!\begin{array}{@{}c@{}} {\sigma}_{u0}^2\kern8.em \\{}\begin{array}{c}{\sigma}_{u01}\kern1em {\sigma}_{u1}^2\kern5.75em \\{}{\sigma}_{u02\kern0.75em }\kern0.5em {\sigma}_{u12}\kern0.75em {\sigma}_{u2}^2\kern3em \\{}0\kern2.25em 0\kern2.25em 0\kern1.5em {\sigma}_{u3}^2\end{array}\!\!\!\!\end{array}\right)\!\right\} \end{align*}

In equation 2, ${y}_{1 hij}$ denotes SBP as measured at (within-clinic) occasion *h* (*h* = 1, …, ${n}_{ij}$) for clinic *i* (*i* = 1, …, ${I}_j$), for individual *j* (*j* = 1, …, J). ${\varepsilon}_{hij}$ refers to the residual within-clinic error in the repeatedly measured exposure, assumed to have constant variance ${\sigma}_{\varepsilon}^2$. This within-clinic measurement error, which we assume is not related to log(LVMI) (${y}_{2j}$), will incorporate both systematic (e.g., white coat effect/habituation) and random error.

Following previous work modeling SBP across these ALSPAC clinics, we allowed for a nonlinear relationship between age and mean SBP by fitting a linear spline with a knot point at 12 years (26, 27). The spline variables were derived as follows (where ${t}_{ij}$ = age in years) (28):

$$\begin{align*} {s}_{1 ij}&=\begin{cases}\kern.5em{t}_{ij}\kern3.8em {t}_{ij}\le 12\\{}\kern.3em 12\kern3.75em {t}_{ij}>12\end{cases}\\[-3pt] {s}_{2 ij}&=\begin{cases}\kern.5em0\kern4.15em {t}_{ij}\le 12\\{}\kern.5em{t}_{ij}-12\kern1.7em {t}_{ij}>12\end{cases} \end{align*}$$

These spline terms were fitted as fixed effects in the mean function for SBP. Because allowing the coefficients of these terms to randomly vary across individuals led to convergence problems in some models, a linear term for age fitted across the whole age range was fitted as a random effect. We assumed that BPV had a linear association with age.

Web Appendix 1 (available at https://doi.org/10.1093/aje/kwaa224) includes estimates from sensitivity analyses designed to 1) investigate the influence of the submodel for log(LVMI) on the random effect estimates (Web Table 1); 2) compare a parameterization modeling clinic mean BP with current models (Web Table 2); 3) check whether restricting the analysis sample to those with LVMI measures had any effect on model for change in SBP, examining possible selection bias (Web Table 3); and 4) investigate sex as the only observed covariate (Web Table 4), for comparison with published findings elsewhere (14, 16).

We used Bayesian estimation via Markov chain Monte Carlo methods in Stan (2.19.1) (29, 30), called from R (R Foundation for Statistical Computing, Vienna, Austria) (31) using the rstan package (32). See Web Appendices 2–3 for examples and further details of estimation.

Ethical approval for the study was obtained from the ALSPAC Ethics and Law Committee and the Local Research Ethics Committees. Informed consent for the use of data collected via questionnaires and clinics was obtained from participants following the recommendations of the ALSPAC Ethics and Law Committee at the time.

## RESULTS

### Cohort description

Two observations at the individual level, 8 at the clinic level, and 1 at the measurement level (36 measurement-level observations in total) were identified as outliers in quantile-quantile plots of residuals from preliminary models and were removed from the data set prior to further analyses. The resulting data set comprised 1,986 individuals attending 10,556 clinic sessions with 19,360 recorded BP measurements.

Table 1 compares the participants included in the analysis with those 12,318 ALSPAC children who were not included (but were recruited in an eligible ALSPAC phase, singletons, and otherwise recorded as alive at 1 year of age). There was evidence that those included were more likely to be female and had mothers who had had fewer children, were more educated, and were healthier than those not included.

**Table 1:** Comparing Characteristics of Children Later Undergoing Echocardiography, and Included in the Model, With Those Not Included in the Model, From the Avon Longitudinal Study of Parents and Children, Southwest England, 1990–2011

**Characteristic**	**Not Included in Model (*n* = 12,318)**			**Included in Model (*n* = 1,986)**		
	**No. in Sample**	**%**	**Mean (SD)**	**No. in Sample**	**%**	**Mean (SD)**
Sex of child						
Female	5,887	47.8		1,090	54.9	
Male	6,431	52.2		896	45.1	
Mother’s education^a^^,^^b^						
CSE/none	2,226	21.7		212	11.5	
Vocational	1,048	10.2		148	8.0	
O level	3,564	34.8		618	33.4	
A level	2,227	21.7		498	27.0	
Degree	1,181	11.5		372	20.1	
Mother’s BMI^a^^,^^b^	9,508		22.9 (3.9)	1,723		22.9 (3.6)
No. of cigarettes smoked per day by mother^a^^,^^b^	9,337		2.5 (5.5)	1,710		1.3 (4.3)
Mother’s age at delivery, years^b^	11,713		27.7 (5.0)	1,895		29.5 (4.6)
Parity^a^^,^^b^	10,745		0.9 (1.0)	1,848		0.7 (0.9)
Child’s birthweight, kg^b^	11,564		3.4 (0.5)	1,873		3.4 (0.5)

Abbreviations: A Level, Advanced Level; BMI, body mass index; CSE, Certificate of Secondary Education; O Level, Ordinary Level; SD, standard deviation.

^a^ Mother’s education: highest educational attainment. Mother’s BMI (weight (kg)/height (m)^2^): at 12 weeks’ gestation. Cigarettes smoked per day by mother: at 32 weeks’ gestation. Parity: mother’s number of previous pregnancies resulting in either a livebirth or a stillbirth.

^b^ Sample sizes for these variables do not sum to that for the total sample due to missing values.

**Figure 1:**
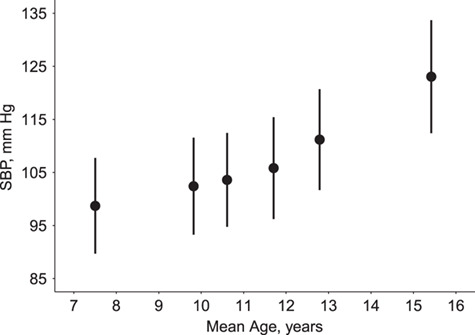
Mean systolic blood pressure (SBP) at clinic against mean age for clinics attended by those participants later undergoing echocardiography, Avon Longitudinal Study of Parents and Children, Southwest England, 1990–2011. Sample sizes (individuals per clinic), for the clinic at the youngest mean age through to the clinic at the oldest mean age, respectively, were *n* = 1,758, *n* = 1,804, *n* = 1,751, *n* = 1,791, *n* = 1,762, and *n* = 1,690. Bars: 1 standard deviation on either side of the mean.

Figure 1 plots mean SBP against mean age for each clinic for those included in the model, indicating sample sizes for each clinic. The mean number of clinics attended was 5.3; the numbers of clinics attended by participants were 1 (*n* = 46), 2 (*n* = 47), 3 (*n* = 68), 4 (*n* = 142), 5 (*n* = 454), and 6 (*n* = 1,229).

### Fixed effects: age and sex

Table 2 summarizes 3-level joint analyses in which both SBP trajectory and log(LVMI) are simultaneously modeled, with age centered around the sample mean of 11.3 years (with some estimates presented as percentage change (33), as indicated; see Web Table 5 for original coefficient estimates). With just age and sex as observed covariates (model A), mean SBP was predicted to increase with age. The rate of increase was similar for boys and girls up to the age of 12 years, with mean BP predicted to increase more steeply for male participants at older ages. With regard to within-individual variance, with each year of age, BPV was predicted to increase by 15.6% (95% credible interval (CrI): 12.9, 18.4). Girls’ BPV was predicted to be 16.8% (95% CrI: 4.5, 30.4) greater than that for boys, across all time points. Age at time of echocardiography was estimated to have a modest negative association with LVMI, and girls were predicted to have smaller values of LVMI (Table 2).

### Fixed effects: adding weight and height

In model B, mean SBP was predicted to increase with greater weight and height. BPV was estimated to be larger with greater log(weight): specifically, for a 10% increase in weight, BPV was predicted to increase by 5.8% (95% CrI: 2.1, 9.6). The estimated effect of height on log(BPV) was negative, with a credible interval including zero (BPV predicted to decrease by −0.4% (95% CrI: −1.4, 0.7) per 1-cm increase in height). Weight had a positive association with LVMI (LVMI predicted to increase by 0.7% (95% CrI: 0.6%, 0.8%) per 1-kg-increase in weight) (Table 2).

### Fixed effects: adding maternal characteristics

Finally, in model C, maternal characteristics were added as covariates in the mean function for SBP (with a slight drop in sample size due to fewer observations for these variables). This predicted a modest positive association of mean SBP with mother’s age at delivery, as well as an inverse association with higher parity and highest educational qualifications (Table 2).

### Random effects

Model C estimated a small negative correlation (*r* = −0.09, 95% CrI: −0.21, 0.03) between mean BP and BP slope (i.e., those with higher mean BP at 11.3 years were predicted to have a smaller increase in their mean BP across age). A large positive correlation (*r* = 0.50, 95% CrI: 0.33, 0.71) was estimated between mean BP and log(BPV) (i.e., individuals with higher mean SBP at 11.3 years also tended to have more fluctuation in their SBP). The correlation between BP slope and log(BPV) was estimated as negative and small (*r* = −0.05, 95% CrI: −0.34, 0.23) (Table 2).

**Figure 2:**
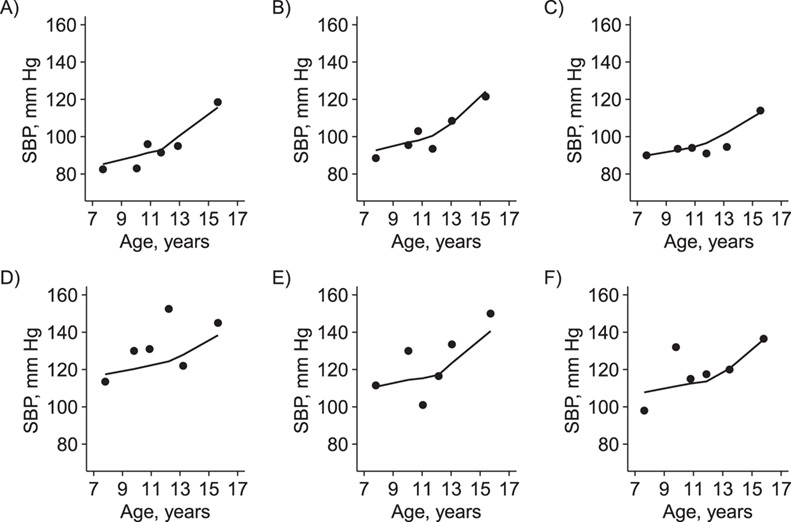
Observed data and predictions for randomly selected individuals according to random effects estimates for blood pressure variability, Avon Longitudinal Study of Parents and Children, Southwest England, 1990–2011. A–C) Three individuals with among the lowest estimates; D–F) 3 individuals with among the highest estimates. Note that a constant has been added to each axis to preserve anonymity. SBP, systolic blood pressure.

The SD of the BPV random effects on the log scale was estimated to have a mean of 0.42 mm Hg (95% CrI: 0.29, 0.53). Figure 2 plots examples from this model of 3 individuals randomly drawn from the 25 with the lowest, and 3 from the 25 with the highest, estimated random effect for log(BPV), with an added constant on both the *x*- and *y*-axis to preserve anonymity, illustrating instances of individual-level patterns of SBP measurements at each end of the observed range of estimated BPV.

**Table 2:** Estimates From Joint Models With Shared Random Effects, Analyzing Systolic Blood Pressure and Log(Left Ventricular Mass Indexed to Height) as Outcomes, Presenting the Posterior Parameter Estimates of the Regression Coefficients, Avon Longitudinal Study of Parents and Children, Southwest England, 1990–2011

**Parameter**	**Model A** ^**a**^		**Model B** ^**a**^		**Model C** ^**a**^	
	**Mean**	**95% CrI**	**Mean**	**95% CrI**	**Mean**	**95% CrI**
Mean SBP: fixed effects						
Intercept	107.95	107.50, 108.40	107.93	107.52, 108.34	108.23	107.29, 109.16
≤12 years^b^	1.71	1.58, 1.85	−0.55	−0.81, −0.30	−0.56	−0.83, −0.30
>12 years^b^	6.00	5.80, 6.21	3.83	3.51, 4.16	3.83	3.49, 4.18
Female	−0.43	−1.02, 0.19	−0.40	−0.96, 0.16	−0.22	−0.81, 0.36
Female × ≤12 years	0.07	−0.11, 0.25	−0.15	−0.33, 0.03	−0.17	−0.36, 0.01
Female × >12 years	−1.99	−2.28, −1.71	−1.26	−1.57, −0.95	−1.25	−1.58, −0.92
Log(weight, kg)			17.01	15.39, 18.64	16.56	14.84, 18.26
Height, cm			0.04	0.00, 0.09	0.06	0.00, 0.11
Maternal characteristics						
Age at delivery, years					0.01	−0.06, 0.09
Parity					−0.17	−0.50, 0.17
Vocational^c^					−0.04	−1.36, 1.30
O Level^c^					0.05	−0.95, 1.03
A Level^c^					−0.64	−1.70, 0.40
Degree^c^					−1.39	−2.50, −0.27
Individual-level random effects for SBP						
SD(mean BP)^d^	6.12	5.88, 6.37	5.37	5.14, 5.60	5.39	5.16, 5.63
SD(BP slope)^d^	0.66	0.55, 0.75	0.64	0.54, 0.73	0.65	0.54, 0.74
SD(log(BPV))^d^	0.41	0.29, 0.51	0.39	0.26, 0.50	0.42	0.29, 0.53
Correlation (mean BP, BP slope)^d^	−0.09	−0.20, 0.02	−0.11	−0.23, 0.00	−0.09	−0.21, 0.03
Correlation (mean BP, log(BPV))^d^	0.52	0.36, 0.71	0.49	0.31, 0.72	0.50	0.33, 0.71
Correlation (BP slope, log(BPV))^d^	−0.06	−0.34, 0.22	−0.04	−0.33, 0.26	−0.05	−0.34, 0.23
BPV: fixed effects						
Intercept	3.22	3.12, 3.32	3.15	3.04, 3.25	3.13	3.02, 3.23
Age, years^e^	15.61	12.87, 18.39	11.32	5.80, 16.98	10.80	5.05, 16.77
Female sex^e^	16.84	4.53, 30.40	17.64	4.66, 31.59	20.06	5.99, 35.46
Log(weight, kg)^f^			5.77	2.10, 9.55	5.24	1.42, 9.20
Height, cm^e^			−0.37	−1.40, 0.70	−0.24	−1.36, 0.89
Residual within-clinic SD for SBP	5.62	5.54, 5.70	5.63	5.54, 5.71	5.61	5.53, 5.70
Mean for log(LVMI): fixed effects						
Intercept	3.36	3.35, 3.37	3.33	3.31, 3.34	3.32	3.31, 3.33
Age, years^e^	−0.07	−2.85, 2.79	−1.65	−4.2, 0.94	−1.94	−4.61, 0.79
Female^e^	−11.00	−12.61, −9.36	−5.42	−7.08, −3.73	−4.95	−6.72, −3.17
Weight, kg^e^			0.69	0.62, 0.76	0.70	0.63, 0.78
mean BP^d^^,^^e^	0.85	0.44, 1.44	0.57	0.09, 1.49	0.59	0.12, 1.37
BP slope^d^^,^^e^	4.38	0.88, 8.61	3.63	0.00, 8.35	3.58	0.01, 7.92
Log(BPV)^d^^,^^f^	−0.61	−2.05, 0.40	−0.82	−3.08, 0.29	−0.78	−2.54, 0.22
Residual SD for log(LVMI)	0.20	0.19, 0.21	0.19	0.17, 0.19	0.19	0.18, 0.19

Abbreviations: A Level, Advanced Level; BP, blood pressure; BPV, (within-individual) blood pressure variability; CrI, credible interval; LVMI, left ventricular mass indexed to height; O Level, Ordinary Level; SBP, systolic blood pressure; SD, standard deviation.

^a^ Model A: adjusted for age and sex (1,986 individuals; 10,556 clinic visits; 19,360 BP measurements); model B, model A with the addition of weight and height (1,986 individuals; 10,556 clinic visits; 19,360 BP measurements); model C: model B with the addition of maternal characteristics (1,813 individuals; 9,693 clinic visits; 17,777 BP measurements).

^b^ Linear spline terms, corresponding to change per year for ages ≤12 years and for ages >12 years.

^c^ Mother’s highest education qualification (referent: Certificate of Secondary Education/none).

^d^ Mean BP denotes the individual-level, between-clinic, random effects for the intercept (at mean age 11.3 years) in the mean function for SBP; BP slope denotes the individual-level, between-clinic, random effects for age in the mean function for SBP; log(BPV) denotes the individual-level, between-clinic, random effects on the log-scale of the within-individual variance (i.e., these are the equivalent of ${u}_0$, ${u}_1$, and ${u}_2$ in equation 2, respectively).

^e^ Estimates presented as percentage change in BPV/LVMI (on natural scale) per 1-unit increase in covariate, calculated (using posterior samples) as (exp(coefficient) − 1) × 100; see Web Table 5 for original coefficient estimates.

^f^ Estimates presented as percentage change in BPV/LVMI (on natural scale) per 10% increase in covariate (on natural scale), calculated (using posterior samples) as ((1.10^coefficient) − 1) × 100; see Web Table 5 for original coefficient estimates.

Table 3 contrasts the estimates of the random effects from model C with those in which the effect of each set of random effects on log(LVMI) is examined without adjustment for the other random effects on log(LVMI), but with the remaining structure of the model otherwise remaining the same; estimates presented as percentage change (33), as indicated (see Web Tables 6–7 for full estimates, including original coefficient estimates). When mean BP is the only random effect included as a predictor of LVMI, higher mean BP is associated with higher LVMI. The same is true when the random slope and the log(BPV) random effects are alternately added as predictors of LVMI. With regard to the log(BPV) predictor, a 10% increase in BPV is predicted to increase LVMI by 0.21% (95% CrI: −0.23, 0.69). When all 3 random effects are included as predictors of LVMI, the estimated association between log(BPV) and LVMI becomes negative and larger in magnitude; specifically, a 10% increase in BPV is estimated to be associated with a decrease in LVMI by −0.78% (95% CrI: −2.54, 0.22). This model also estimated a positive association of mean BP with LVMI (a 1-mm-Hg increase in BP at the sample mean age was predicted to increase LVMI by 0.59%, 95% CrI: 0.12, 1.37) and of change in mean BP with LVMI (a 1-mm-Hg increase in the slope was predicted to increase LVMI by mean = 3.58%, 95% CrI: 0.01, 7.92).

### Sensitivity analyses

As Web Appendix 1 further discusses, a range of sensitivity analyses yielded substantively similar estimates to the models presented here.

## DISCUSSION

Using data from a prospective longitudinal birth cohort study based in the United Kingdom, we used a joint modeling approach to examine the factors associated with visit-to-visit BPV across childhood and the association of both mean BP and BPV with an established biomarker of target organ damage, LVMI, in early adulthood. BPV was estimated to be larger (i.e., individuals’ SBP was estimated to fluctuate more greatly) at older ages, in girls, and at greater body weights. BPV also had a large, positive correlation with mean BP (at the sample mean age of 11.3 years) but only a very small correlation with the rate of change in BP (slope) across age. In a model that did not adjust for the effect of mean BP and its slope on LVMI, BPV was estimated to have a weak positive association with LVMI. When mean BP and its slope were also included as covariates, however, the direction of association between BPV and LVMI changed and the magnitude of the estimate increased. These models further estimated a positive association of mean BP with LVMI and of change in mean BP with LVMI.

While we are not aware of other studies of subjects in this age range that have reported on the effect of weight and height on BPV, the effect of age, sex, and race have been examined. Investigating BPV in 8- to18-year-old children in Massachusetts, Rosner et al. (16) fitted variance components models to BP measurements taken within and across visits (up to a maximum of 16 visits over 4 years) and subsequently examined the effect of age, sex, and BP level on these 2 variance components in 3-way analyses of variance. They found “no meaningful effects” of these covariates on variability of SBP (children with higher BP level had significantly more within-individual variability than those with lower BP, but this difference was judged small; correlation coefficient not reported). More recently, in an analysis of the Bogalusa Heart Study in the United States, Chen et al. (14) measured visit-to-visit BPV in children aged 4–19 years by taking the SD of 4–8 measurements of clinic mean SBP from visits scheduled every 2–3 years. Boys were estimated to have significantly higher SD(SBP) than girls, and SD(SBP) was estimated to be significantly greater for Blacks than for Whites. Our supplementary analyses (Web Table 4) also indicated that girls were estimated to have lower BPV than boys but not once the effect of time-varying covariates were adjusted for. In addition, and in keeping with our findings, Chen et al. (14) found a positive correlation (*r* = 0.15) between mean childhood BP level and SD(SBP). Such a positive correlation has been characterized in studies of adults, too (8, 34).

While several studies have found a positive relationship between adult BPV, independent of mean BP, and later cardiovascular events such as stroke (8) and all-cause mortality (34), evidence for the relationship between BPV and target organ damage (as indicated by left ventricular hypertrophy, for example) is more equivocal (35, 36). A recent review described contrasting results with regard to the association of visit-to-visit BPV and cardiovascular outcomes, suggesting that differences between (adult) study populations, such as underlying cardiovascular risk, might be an important determinant of such heterogeneous findings (7). With regard to the relationship between childhood BPV and adult biomarkers, Chen et al. (14) found childhood visit-to-visit SD (SBP) to be significantly associated with adult hypertension. This remained the case after adjusting for mean childhood SBP, although elevated childhood SBP levels were found to be more predictive of adult hypertension than childhood BPV. We have previously demonstrated that, in 17-year-olds, higher body mass index is causally related to higher LVMI, suggesting that there is meaningful variation in cardiac structure measurements in early adulthood that is likely to track across life and relate to later life cardiovascular health (37). Nevertheless, in the current study only a weak positive association between childhood BPV and LVMI in early adulthood was found, and this did not persist once childhood mean BP had been adjusted for. In fact, within strata of mean childhood SBP, higher BPV was associated with smaller values of LVMI. Whether it is appropriate to adjust for mean BP when assessing the association of BPV with LVMI somewhat depends on whether one has a causal model in mind that conceptualizes mean BP as causing both BPV and LVMI (in which case, adjustment for mean BP as a confounder might be appropriate) or whether no causal relationship is proposed between mean BP and BPV (i.e., other, unknown, factors cause mean BP and BPV, and each in turn has a causal relationship with later LVMI), in which case it might not be appropriate to adjust for mean BP. If the purpose is prediction, on the other hand, then by simultaneously estimating the effect of mean BP, its slope, and BPV on the later outcome, we allow for a more complete appraisal of the association of repeatedly measured BP with LVMI. Note that whether mean BP is adjusted for or not, the association of BPV with LVMI was estimated to be small, with appreciable uncertainty as to whether it was nonzero, or indeed of the opposite sign.

**Table 3:** Posterior Parameter Estimates From Joint Models in Which Mean Blood Pressure, Blood Pressure Slope, and Log(Blood Pressure Variability) Are Included as Exposures for Log(Left Ventricular Mass Indexed to Height)^a^, From the Avon Longitudinal Study of Parents and Children, Southwest England, 1990–2011

**Parameter**	**Mean BP Only**		**BP Slope Only**		**Log(BPV) Only**		**Mean BP, BP Slope, Log(BPV) All Included**	
	**Mean, %**	**95% CrI**	**Mean, %**	**95% CrI**	**Mean, %**	**95% CrI**	**Mean, %**	**95% CrI**
Mean BP^b^^,^^c^	0.19	0.00, 0.39					0.59	0.12, 1.37
BP slope^b^^,^^c^			2.40	−0.51, 5.56			3.58	0.01, 7.92
Log(BPV)^b^^,^^d^					0.21	−0.23, 0.69	−0.78	−2.54, 0.22

Abbreviations: BP, blood pressure; BPV, blood pressure variability; CrI, credible interval; LVMI, left ventricular mass indexed to height.

^a^ These are selected parameter estimates from these models; see Web Tables 6–7 for full models.

^b^ Mean BP denotes the individual-level, between-clinic, random effects for the intercept (at mean age 11.3 years) in the mean function for SBP; BP slope denotes the individual-level, between-clinic, random effects for age in the mean function for SBP; log(BPV) denotes the individual-level, between-clinic, random effects on the log-scale of the within-individual variance (i.e., these are the equivalent of ${u}_0$, ${u}_1$, and ${u}_2$ in equation 2, respectively).

^c^ Estimates presented as percentage change in LVMI (on natural scale) per 1-unit change in covariate, calculated (using posterior samples) as (exp(coefficient) − 1) × 100; see Web Table 7 for original coefficient estimates.

^d^ Estimates presented as percentage change in LVMI (on natural scale) per 10% increase in covariate (on natural scale), calculated (using posterior samples) as ((1.10^coefficient) − 1) × 100; see Web Table 7 for original coefficient estimates.

Our results also indicated that mean BP (at the sample mean age) and change in mean BP were both positively associated with LVMI in early adulthood. Analyses of the Georgia Stress and Heart Study and the Bogalusa Heart Study have also found mean BP in childhood, and its slope from childhood to young adulthood and in adolescence, to be positively associated with left ventricular hypertrophy in adulthood (38–40).

If we assume that selection into our analysis sample depends only on measured variables such as sex of child and education and age of mother, then their inclusion as covariates will result in unbiased models. If, having conditioned on such covariates, inclusion in our analysis sample is related to the exposure (SBP) and outcome (LVMI), then there will be bias. A priori, this seems relatively unlikely, however (e.g., such biomarkers are not readily observable in a manner that would typically influence one’s participation in a study). While there were fewer observations for the maternal (compared with child-based) characteristics, the proportion of missing data was relatively modest (8%–9%), and estimates for parameters common to each model were substantively similar.

Selection bias could also arise from people missing values for specific measures. Again, this will only cause bias if the probability of the measure being missing depends on its (unobserved) value, having conditioned on the observed values of the exposure, outcome, and covariates included in the models. This again seems relatively unlikely, however, given that participants are not typically privy to such biomarkers in a manner that might influence attendance.

The general class of model we have used could be employed to examine within-person variability in any repeatedly measured exposure, relating it to later individual-level outcomes of interest within a joint model (e.g., the association between glycemic variability and mortality (41), variability in gait and risk of falling (42), and variation in prostate specific antigen and prostate volume (43)). We have focused on within-individual variability, but interest might lie in characterizing within-clinic (interindividual) variability instead. Also, individual-level outcomes (i.e., ${y}_{2j}$, in equations 1 and 2) that are binary, or time-to-event data (3), etc., can be modeled (with appropriate changes to distributional assumptions). Furthermore, the repeatedly measured exposure might instead be intensively sampled, with many observations per person (24), adjusting for autocorrelation, if appropriate.

While fitting joint models involves greater computational burden and complexity compared with a 2-stage approach, the latter is known to introduce bias (3). Note, though, that a recent methodological development of the 2-stage approach preserves uncertainty in estimates (44). A 2-stage approach might appear more logical in cases where it is not feasible for the later outcome to influence the earlier longitudinal process, due to fears over allowing the future to cause the past (44, 45). The joint modeling framework, however, uses future measurements to improve the model for the past measurements, rather than to cause the past measurements. This is the case in a wide range of model types and applications (5, 46, 47), such as a growth curve model, where adding later waves revises estimates of true values for earlier occasions.

We have supplied example code to demonstrate how these models can be fitted using a Bayesian framework, with endeavors to fit such models being actively extended to other software packages (48). Joint models have recently been employed to examine within-individual variability (3). We further extend these by investigating the association of time-varying covariates with within-individual variability, allowing us to examine residual BPV unexplained by known factors, and also by distinguishing between-clinic from within-clinic variability. As such, the joint modeling approach we have introduced offers a very flexible method of exploring the factors associated with within-individual variability. 

## Supplementary Material

Web_Material_kwaa224
