# Supplementary material for: Joint Modeling of Individual Trajectories, Within-Individual Variability, and a Later Outcome: Systolic Blood Pressure Through Childhood and Left Ventricular Mass in Early Adulthood
Source: Am J Epidemiol. 2020 Oct 15;190(4):652–62. doi: 10.1093/aje/kwaa224 (PMC8024053; doi:10.1093/aje/kwaa224)
Supplement: Web_Material_kwaa224 [file web_material_kwaa224.pdf]

Web Material

Joint Modeling of Individual Trajectories, Within-Individual Variability, and a Later Outcome: Systolic Blood Pressure Through Childhood and Left Ventricular Mass in Early Adulthood

Richard M.A. Parker, George Leckie, Harvey Goldstein, Laura D. Howe, Jon Heron, Alun D. Hughes, David M. Phillippo, and Kate Tilling

Table of Contents

Web Appendix 1 2

Web Table 1 2

Web Table 2. 5

Web Table 3 9

Web Table 4 12

Web Table 5 13

Web Table 6 16

Web Table 7 19

Web Appendix 2 23

Web Appendix 3 45

References 46

Web Appendix 1

Web Appendix 1 includes estimates from: (a) univariate outcome (SBP) models analysing the same sample and covariates presented below to investigate the influence of the submodel for log(LVMI) on the random effect estimates (Web Table 1); (b) models in which the mean of the BP measurements taken for each individual at each clinic was analysed as the repeatedly-measured exposure, in 2-level models akin to equation 1 (main manuscript), to compare modelling clinic BP (Web Table 2); (c) a univariate outcome model of SBP, allowing for complex BPV, fitted to all those for whom at least one mean SBP measurement, from at least one of the six clinics, is available (regardless of whether later had LVMI estimated) to check whether restricting the analysis sample to those with LVMI measures has any effect on model for change in SBP, and therefore examining possible selection bias (Web Table 3); (d) a model in which sex is the only observed covariate included (Web Table 4), for comparison with published findings elsewhere (1, 2); and (e) full model estimates from the joint models presented in the main manuscript (Web Tables 5-7).

Web Table 1

As Web Table 1 indicates, univariate outcome (SBP) models analysing the same sample and covariates as in the main manuscript yielded substantively similar estimates to joint models, indicating modest influence of the submodel for the subsequent outcome (log(LVMI)) on the random effect estimates.

Web Table 1: estimates from univariate outcome models, analysing SBP as the outcome, presenting the mean and credible intervals for the posterior parameter estimates of the regression coefficients (except where indicated).

Model	Suppl Model A: Age & Sex			Suppl Model B: Adding weight and height			Suppl Model C: Adding maternal characteristics		
N individuals	1,986			1,986			1,813		
N clinic visits	10,556			10,556			9,693		
N BP measurements	19,360			19,360			17,777		
	Mean	2.5%	97.5%	Mean	2.5%	97.5%	Mean	2.5%	97.5%
Mean SBP: fixed effects									
Intercept	107.96	107.51	108.40	107.96	107.55	108.37	108.22	107.28	109.14
≤12 years[a]	1.71	1.58	1.84	-0.49	-0.73	-0.24	-0.48	-0.75	-0.22
>12 years[a]	6.02	5.81	6.22	3.94	3.62	4.26	3.95	3.62	4.29
Female	-0.43	-1.05	0.18	-0.45	-1.01	0.11	-0.28	-0.86	0.30
Female*≤12 years	0.07	-0.11	0.25	-0.14	-0.32	0.04	-0.16	-0.34	0.03
e Female*>12 years	-2.00	-2.28	-1.71	-1.33	-1.63	-1.02	-1.32	-1.64	-1.00
log(Weight, kg)				17.44	15.84	19.05	17.04	15.37	18.72
Height (cm)				0.02	-0.02	0.07	0.03	-0.02	0.08
Maternal characteristics									
Age at delivery (years)							0.01	-0.06	0.08
Parity							-0.17	-0.50	0.16
Highest ed.: vocational[b]							0.03	-1.31	1.35

Highest ed.: O-level[b]							0.09	-0.90	1.11
Highest ed.: A-level[b]							-0.61	-1.65	0.44
Highest ed.: Degree[b]							-1.32	-2.42	-0.20
Individual-level random effects for SBP									
SD(mean BP)[c]	6.12	5.88	6.37	5.36	5.14	5.59	5.38	5.15	5.62
SD(BP slope)[c]	0.66	0.56	0.76	0.65	0.55	0.74	0.65	0.55	0.75
SD(log(BPV))[c]	0.43	0.33	0.53	0.41	0.30	0.51	0.44	0.32	0.54
Cor(mean BP, BP slope)[c]	-0.09	-0.20	0.02	-0.11	-0.23	0.00	-0.09	-0.21	0.03
Cor(mean BP, log(BPV))[c]	0.51	0.36	0.67	0.47	0.30	0.65	0.48	0.32	0.66
Cor(BP slope, log(BPV))[c]	-0.05	-0.31	0.21	-0.05	-0.32	0.23	-0.05	-0.32	0.21
BPV: fixed effects									
Intercept	3.21	3.11	3.31	3.14	3.03	3.24	3.11	3.00	3.22
Age (years)[d]	15.59%	12.85%	18.40%	10.60%	5.35%	16.14%	10.03%	4.39%	15.85%
Female[d]	17.29%	4.71%	30.83%	18.62%	5.41%	33.04%	21.05%	6.85%	36.63%
log(Weight, kg)[e]				5.43%	1.78%	9.12%	4.79%	0.99%	8.65%
Height (cm)[d]				-0.18%	-1.19%	0.82%	-0.02%	-1.09%	1.06%
Residual within-clinic SD for SBP	5.62	5.54	5.70	5.63	5.54	5.71	5.62	5.53	5.70

[a] Linear spline terms, corresponding to change per year for age ≤ 12, and for age >12, respectively.

[b] Reference category for mother's highest education qualification: CSE / None.

[c] mean BP denotes the individual-level, between-clinic, random effects for the intercept (at mean age 11.3 years) in the mean function for SBP; BP slope denotes the individual-level, between-clinic, random effects for age in the mean function for SBP; log(BPV) denotes the individual-level, between-clinic, random effects on the log-scale of the within-individual variance.

[d] estimates presented as percentage change in BPV (on natural scale) per 1-unit increase in covariate (3), calculated (using posterior samples) as (exp(coefficient) - 1) * 100.

[e] estimates presented as percentage change in BPV (on natural scale) per 1-unit increase in covariate (3), calculated (using posterior samples) as (exp(coefficient) - 1) * 100.

Web Table 2.

As Web Table 2 indicates, when modelling mean clinic BP as the outcome (in two-level models), the estimated associations were substantively similar to those found in the three-level models (analysing the same sample) in the main manuscript, although with some differences. For example, the SD of the log(BPV) random effects is estimated to be smaller in the two-level models, indicating that individuals are estimated to differ to a lesser degree in how erratic their BP measurements are when mean clinic BP is modelled as the outcome. In addition, the fixed intercept in the log(BPV) function is larger in the two-level models, indicating that within-individual variance is estimated to be greater, whilst the estimates of the fixed effects in that function are smaller.

Web Table 2: Estimates from 2-level joint models with shared random effects, analysing SBP and log(LVMI) as outcomes, presenting the mean and credible intervals for the posterior parameter estimates of the regression coefficients (except where indicated). The repeatedly-measured outcome is the mean of the SBP measurements taken for each individual at each clinic.

Model	mean BP only			BP slope only			log(BPV) only			mean BP, BP slope, log(BPV) all included		
N individuals	1,813			1,813			1,813			1,813		
N clinic visits	9,693			9,693			9,693			9,693		
	Mean	2.5%	97.5%	Mean	2.5%	97.5%	Mean	2.5%	97.5%	Mean	2.5%	97.5%
Mean SBP: fixed effects												
Intercept	107.84	106.90	108.77	107.81	106.87	108.75	107.82	106.88	108.74	107.83	106.90	108.76
≤12 years[a]	-0.49	-0.76	-0.22	-0.44	-0.70	-0.18	-0.45	-0.71	-0.19	-0.51	-0.78	-0.25
>12 years[a]	3.91	3.57	4.24	3.97	3.64	4.30	3.96	3.62	4.30	3.86	3.52	4.20
Female	-0.17	-0.76	0.42	-0.19	-0.77	0.39	-0.19	-0.77	0.39	-0.14	-0.73	0.44
Female*≤12 years	-0.17	-0.36	0.02	-0.16	-0.35	0.03	-0.16	-0.35	0.03	-0.18	-0.37	0.01
Female*>12 years	-1.28	-1.60	-0.96	-1.32	-1.64	-1.00	-1.31	-1.64	-0.99	-1.25	-1.57	-0.92
log(Weight, kg)	16.37	14.66	18.07	16.53	14.85	18.21	16.55	14.85	18.25	16.13	14.42	17.86
Height (cm)	0.05	0.00	0.10	0.04	-0.01	0.08	0.04	-0.01	0.09	0.06	0.01	0.11
Maternal characteristics												
Age at delivery (years)	0.02	-0.06	0.09	0.01	-0.06	0.08	0.01	-0.06	0.08	0.02	-0.06	0.09
Parity	-0.16	-0.49	0.17	-0.17	-0.50	0.17	-0.16	-0.50	0.17	-0.16	-0.49	0.17

Highest ed.: vocational[b]	-0.06	-1.40	1.25	-0.01	-1.33	1.31	-0.02	-1.35	1.30	-0.08	-1.41	1.24
Highest ed.: O-level[b]	0.11	-0.86	1.10	0.16	-0.83	1.14	0.15	-0.84	1.14	0.10	-0.89	1.09
Highest ed.: A-level[b]	-0.59	-1.62	0.45	-0.56	-1.60	0.48	-0.56	-1.61	0.48	-0.60	-1.63	0.42
Highest ed.: Degree[b]	-1.37	-2.50	-0.28	-1.32	-2.43	-0.22	-1.32	-2.42	-0.20	-1.39	-2.48	-0.29
Individual-level random effects for SBP												
SD(mean BP)[c]	5.35	5.12	5.59	5.36	5.12	5.59	5.35	5.12	5.59	5.35	5.12	5.59
SD(BP slope)[c]	0.61	0.49	0.71	0.60	0.49	0.71	0.60	0.49	0.71	0.60	0.48	0.70
SD(log(BPV))[c]	0.27	0.21	0.33	0.27	0.21	0.33	0.26	0.19	0.33	0.26	0.19	0.33
Cor(mean BP, BP slope)[c]	-0.09	-0.22	0.04	-0.06	-0.20	0.07	-0.09	-0.22	0.04	-0.09	-0.23	0.04
Cor(mean BP, log(BPV))[c]	0.49	0.34	0.66	0.49	0.34	0.66	0.51	0.35	0.70	0.51	0.34	0.70
Cor(BP slope, log(BPV))[c]	-0.09	-0.36	0.19	-0.11	-0.38	0.16	-0.07	-0.35	0.22	-0.09	-0.39	0.20
BPV: fixed effects												
Intercept	3.74	3.68	3.80	3.74	3.68	3.80	3.74	3.68	3.80	3.75	3.69	3.80
Age (years)[d]	5.36%	1.94%	8.80%	5.44%	2.03%	8.92%	5.36%	1.93%	8.84%	6.13%	2.65%	9.74%
Female[d]	12.76%	4.73%	21.10%	12.85%	4.83%	21.27%	12.87%	4.94%	21.17%	12.20%	4.31%	20.54%
log(Weight, kg)[e]	3.30%	1.03%	5.65%	3.34%	1.05%	5.68%	3.25%	0.96%	5.62%	3.68%	1.37%	6.07%
Height (cm)[d]	-0.16%	-0.82%	0.51%	-0.19%	-0.86%	0.48%	-0.14%	-0.82%	0.54%	-0.35%	-1.06%	0.35%
Mean for log(LVMI): fixed effects												
Intercept	3.32	3.31	3.33	3.32	3.31	3.33	3.32	3.31	3.33	3.32	3.31	3.33

Age (years)[d]	-1.97%	-4.65%	0.78%	-2.10%	-4.74%	0.62%	-2.04%	-4.73%	0.69%	-1.94%	-4.63%	0.80%
Female[d]	-4.99%	-6.76%	-3.20%	-4.97%	-6.75%	-3.21%	-5.00%	-6.76%	-3.22%	-4.95%	-6.72%	-3.16%
Weight (kg)[d]	0.70%	0.63%	0.77%	0.70%	0.63%	0.77%	0.70%	0.62%	0.77%	0.70%	0.63%	0.78%
mean BP[c,d]	0.20%	0.00%	0.40%							0.63%	0.17%	1.31%
BP slope[c,d]				3.00%	-0.54%	7.07%				4.15%	-0.12%	9.52%
log(BPV)[c,e]							0.25%	-0.45%	1.00%	-1.33%	-3.59%	0.17%
Residual SD for log(LVMI)	0.19	0.18	0.20	0.19	0.18	0.20	0.19	0.18	0.20	0.19	0.18	0.19

[a] Linear spline terms, corresponding to change per year for age ≤ 12, and for age >12, respectively.

[b] Reference category for mother's highest education qualification: CSE / None.

[c] mean BP denotes the individual-level, between-clinic, random effects for the intercept (at mean age 11.3 years) in the mean function for SBP; BP slope denotes the individual-level, between-clinic, random effects for age in the mean function for SBP; log(BPV) denotes the individual-level, between-clinic, random effects on the log-scale of the within-individual variance.

[d] estimates presented as percentage change in BPV / LVMI (on natural scale) per 1-unit increase in covariate (3), calculated (using posterior samples) as (exp(coefficient) - 1) * 100.

[e] estimates presented as percentage change in BPV / LVMI (on natural scale) per 10% increase in covariate (on natural scale) (3), calculated (using posterior samples) as ((1.10^coefficient) - 1) * 100.

Web Table 3

As Web Table 3 indicates, a univariate outcome model of SBP fitted to all those for whom at least one mean SBP measurement, from at least one of the six clinics, was available (i.e. regardless of whether the individual later had their LVMI estimated) found associations broadly in keeping with those in the smaller subset modelled in the main manuscript. The SD of the log(BPV) random effects was a little higher in this larger sample, and age and sex were estimated to have smaller associations with log(BPV), and weight and height larger associations, as well.

Web Table 3: Estimates from univariate outcome models, analysing SBP as the outcome, presenting the mean and credible intervals for the posterior parameter estimates of the regression coefficients (except where indicated).

Model	Age & Sex			Adding weight and height			Adding maternal characteristics		
N individuals	9,447			9,447			8,232		
N clinic visits	40,345			40,345			35,998		
N BP measurements	73,749			73,749			65,805		
	Mean	2.5%	97.5%	Mean	2.5%	97.5%	Mean	2.5%	97.5%
Mean SBP: fixed effects									
Intercept	107.29	107.08	107.50	107.32	107.14	107.50	107.68	107.29	108.08
≤12 years[a]	1.59	1.53	1.66	-0.59	-0.71	-0.47	-0.60	-0.72	-0.47
>12 years[a]	5.86	5.75	5.97	3.74	3.58	3.91	3.77	3.60	3.94
Female	-0.42	-0.71	-0.13	-0.55	-0.81	-0.29	-0.47	-0.75	-0.19
Female*≤12 years	0.20	0.11	0.29	-0.03	-0.13	0.06	-0.03	-0.13	0.06

Female*>12 years	-1.90	-2.06	-1.74	-1.23	-1.39	-1.06	-1.24	-1.42	-1.07
log(Weight, kg)				17.64	16.85	18.42	17.45	16.61	18.31
Height (cm)				0.02	0.00	0.05	0.03	0.00	0.05
Maternal characteristics									
Age at delivery (years)							-0.01	-0.04	0.03
Parity							-0.13	-0.29	0.02
Highest ed.: vocational[b]							0.05	-0.54	0.64
Highest ed.: O-level[b]							-0.23	-0.67	0.20
Highest ed.: A-level[b]							-0.73	-1.19	-0.27
Highest ed.: Degree[b]							-1.17	-1.70	-0.65
Individual-level random effects for SBP									
SD(mean BP)[c]	6.19	6.07	6.32	5.28	5.16	5.39	5.25	5.13	5.37
SD(BP slope)[c]	0.73	0.68	0.79	0.71	0.65	0.76	0.71	0.65	0.76
SD(log(BPV))[c]	0.54	0.50	0.59	0.53	0.48	0.58	0.54	0.48	0.59
Cor(mean BP, BP slope)[c]	-0.04	-0.10	0.02	-0.06	-0.12	0.00	-0.04	-0.10	0.02
Cor(mean BP, log(BPV))[c]	0.49	0.43	0.55	0.42	0.36	0.49	0.43	0.36	0.50
Cor(BP slope, log(BPV))[c]	-0.09	-0.21	0.02	-0.11	-0.22	0.01	-0.09	-0.21	0.03
BPV: fixed effects									
Intercept	3.24	3.19	3.29	3.18	3.13	3.23	3.17	3.11	3.22
Age (years)[d]	13.06%	11.60%	14.56%	8.60%	5.72%	11.53%	8.00%	4.93%	11.10%

Female[d]	12.88%	6.18%	19.79%	11.68%	5.07%	18.59%	12.86%	5.62%	20.47%
log(Weight, kg)[e]				8.36%	6.41%	10.33%	8.57%	6.43%	10.70%
Height (cm)[d]				-0.96%	-1.50%	-0.41%	-0.93%	-1.51%	-0.35%
Residual within-clinic SD for SBP	5.50	5.46	5.54	5.51	5.47	5.55	0.06	5.46	5.55

[a] Linear spline terms, corresponding to change per year for age ≤ 12, and for age >12, respectively.

[b] Reference category for mother's highest education qualification: CSE / None.

[c] mean BP denotes the individual-level, between-clinic, random effects for the intercept (at mean age 11.3 years) in the mean function for SBP; BP slope denotes the individual-level, between-clinic, random effects for age in the mean function for SBP; log(BPV) denotes the individual-level, between-clinic, random effects on the log-scale of the within-individual variance.

[d] estimates presented as percentage change in BPV (on natural scale) per 1-unit increase in covariate (3), calculated (using posterior samples) as (exp(coefficient) - 1) * 100.

[e] estimates presented as percentage change in BPV (on natural scale) per 10% increase in covariate (on natural scale) (3), calculated (using posterior samples) as ((1.10^coefficient) - 1) * 100.

Web Table 4

As Web Table 4 indicates, when sex is the only covariate (analysing the same sample as models presented in main manuscript), females' BPV was predicted to be -28.31% (-33.09%, -23.21%) lower than that for males.

Web Table 4: Estimates from univariate outcome model, analysing SBP as the outcome, with sex as the only covariate, presenting the mean and credible intervals for the posterior parameter estimates of the regression coefficients (except where indicated).

Model	Sex		
N individuals	1,986		
N clinic visits	10,556		
N BP measurements	19,360		
	Mean	2.5%	97.5%
Mean SBP: fixed effects			
Intercept	107.63	107.16	108.11
Female	-0.35	-0.97	0.28
Individual-level random effects for SBP			
SD(mean BP)[a]	4.95	4.64	5.27
SD(log(BPV))[a]	0.12	0.07	0.18
Cor(mean BP, log(BPV))[a]	0.80	0.54	0.97
BPV: fixed effects			

Intercept	4.86	4.81	4.91
Female[b]	-28.31%	-33.09%	-23.21%
Residual within-clinic SD			
for SBP	5.61	5.53	5.69

[a] mean BP denotes the individual-level, between-clinic, random effects for the intercept (at mean age 11.3 years) in the mean function for SBP; BP slope denotes the individual-level, between-clinic, random effects for age in the mean function for SBP; log(BPV) denotes the individual-level, between-clinic, random effects on the log-scale of the within-individual variance.

[b] estimates presented as percentage change in BPV (on natural scale) per 1-unit increase in covariate (3), calculated (using posterior samples) as (exp(coefficient) - 1) * 100.

Web Table 5

Web Table 5: Estimates from the joint models presented in Table 2 in the main manuscript, but with original, non-transformed, regression coefficients presented for all parameter estimates.

Model	A: Age & Sex			B: Adding weight and height			C: Adding maternal characteristics		
N individuals	1,986			1,986			1,813		
N clinic visits	10,556			10,556			9,693		
N BP measurements	19,360			19,360			17,777		
	Mean	2.5%	97.5%	Mean	2.5%	97.5%	Mean	2.5%	97.5%

Mean SBP: fixed effects									
Intercept	107.95	107.50	108.40	107.93	107.52	108.34	108.23	107.29	109.16
≤12 years[a]	1.71	1.58	1.85	-0.55	-0.81	-0.30	-0.56	-0.83	-0.30
>12 years[a]	6.00	5.80	6.21	3.83	3.51	4.16	3.83	3.49	4.18
Female	-0.43	-1.02	0.19	-0.40	-0.96	0.16	-0.22	-0.81	0.36
Female*≤12 years	0.07	-0.11	0.25	-0.15	-0.33	0.03	-0.17	-0.36	0.01
Female*>12 years	-1.99	-2.28	-1.71	-1.26	-1.57	-0.95	-1.25	-1.58	-0.92
log(Weight, kg)				17.01	15.39	18.64	16.56	14.84	18.26
Height (cm)				0.04	0.00	0.09	0.06	0.00	0.11
Maternal characteristics									
Age at delivery (years)							0.01	-0.06	0.09
Parity							-0.17	-0.50	0.17
Highest ed.: vocational[b]							-0.04	-1.36	1.30
Highest ed.: O-level[b]							0.05	-0.95	1.03
Highest ed.: A-level[b]							-0.64	-1.70	0.40
Highest ed.: Degree[b]							-1.39	-2.50	-0.27
Individual-level random effects for SBP									
SD(mean BP)[c]	6.12	5.88	6.37	5.37	5.14	5.60	5.39	5.16	5.63
SD(BP slope)[c]	0.66	0.55	0.75	0.64	0.54	0.73	0.65	0.54	0.74
SD(log(BPV))[c]	0.41	0.29	0.51	0.39	0.26	0.50	0.42	0.29	0.53

Cor(mean BP, BP slope)[c]	-0.09	-0.20	0.02	-0.11	-0.23	0.00	-0.09	-0.21	0.03
Cor(mean BP, log(BPV))[c]	0.52	0.36	0.71	0.49	0.31	0.72	0.50	0.33	0.71
Cor(BP slope, log(BPV))[c]	-0.06	-0.34	0.22	-0.04	-0.33	0.26	-0.05	-0.34	0.23
BPV: fixed effects									
Intercept	3.22	3.12	3.32	3.15	3.04	3.25	3.13	3.02	3.23
Age (years)	0.14	0.12	0.17	0.11	0.06	0.16	0.10	0.05	0.15
Female	0.15	0.04	0.27	0.16	0.05	0.27	0.18	0.06	0.30
log(Weight, kg)				0.59	0.22	0.96	0.53	0.15	0.92
Height (cm)				0.00	-0.01	0.01	0.00	-0.01	0.01
Residual within-clinic SD									
for SBP	5.62	5.54	5.70	5.63	5.54	5.71	5.61	5.53	5.70
Mean for log(LVMI): fixed effects									
Intercept	3.36	3.35	3.37	3.33	3.31	3.34	3.32	3.31	3.33
Age (years)	0.00	-0.03	0.03	-0.02	-0.04	0.01	-0.02	-0.05	0.01
Female	-0.12	-0.13	-0.10	-0.06	-0.07	-0.04	-0.05	-0.07	-0.03
Weight (kg)				0.01	0.01	0.01	0.01	0.01	0.01
mean BP[c]	0.01	0.00	0.01	0.01	0.00	0.01	0.01	0.00	0.01
BP slope[c]	0.04	0.01	0.08	0.04	0.00	0.08	0.03	0.00	0.08
log(BPV)[c]	-0.06	-0.22	0.04	-0.09	-0.33	0.03	-0.08	-0.27	0.02
Residual SD for log(LVMI)	0.20	0.19	0.21	0.19	0.17	0.19	0.19	0.18	0.19

[a] Linear spline terms, corresponding to change per year for age ≤ 12, and for age >12, respectively.

[b] Reference category for mother's highest education qualification: CSE / None.

[c] mean BP denotes the individual-level, between-clinic, random effects for the intercept (at mean age 11.3 years) in the mean function for SBP; BP slope denotes the individual-level, between-clinic, random effects for age in the mean function for SBP; log(BPV) denotes the individual-level, between-clinic, random effects on the log-scale of the within-individual variance; i.e. these are the equivalent of u_0 , u_1 and u_2 in equation 2, respectively.

Web Table 6

Web Table 6: Full estimates from the joint models presented in Table 3 in the main manuscript: i.e. estimates from joint models in which mean BP, BP slope, and log(BPV) are alternately included as exposures for log(LVMI). The mean and credible intervals for the posterior parameter estimates of the regression coefficients (except where indicated).

Model	mean BP only			BP slope only			log(BPV) only			mean BP, BP slope, log(BPV) all included		
N individuals	1,813			1,813			1,813			1,813		
N clinic visits	9,693			9,693			9,693			9,693		
N BP measurements	17,777			17,777			17,777			17,777		
	Mean	2.5%	97.5%	Mean	2.5%	97.5%	Mean	2.5%	97.5%	Mean	2.5%	97.5%
Mean SBP: fixed effects												
Intercept	108.23	107.28	109.18	108.23	107.29	109.17	108.21	107.26	109.14	108.23	107.29	109.16

≤12 years[a]	-0.54	-0.80	-0.27	-0.49	-0.75	-0.23	-0.51	-0.77	-0.25	-0.56	-0.83	-0.30
>12 years[a]	3.88	3.54	4.23	3.94	3.60	4.28	3.92	3.58	4.26	3.83	3.49	4.18
Female	-0.25	-0.83	0.33	-0.29	-0.87	0.30	-0.27	-0.85	0.32	-0.22	-0.81	0.36
Female*≤12 years	-0.17	-0.35	0.02	-0.16	-0.34	0.03	-0.16	-0.35	0.02	-0.17	-0.36	0.01
Female*>12 years	-1.29	-1.61	-0.96	-1.32	-1.64	-1.00	-1.31	-1.63	-0.98	-1.25	-1.58	-0.92
log(Weight, kg)	16.79	15.08	18.51	16.95	15.24	18.64	16.93	15.22	18.64	16.56	14.84	18.26
Height (cm)	0.05	-0.01	0.10	0.03	-0.02	0.08	0.04	-0.01	0.09	0.06	0.00	0.11
Maternal characteristics												
Age at delivery (years)	0.01	-0.06	0.08	0.01	-0.06	0.08	0.01	-0.06	0.08	0.01	-0.06	0.09
Parity	-0.16	-0.50	0.17	-0.17	-0.51	0.17	-0.17	-0.50	0.17	-0.17	-0.50	0.17
Highest ed.: vocational[b]	-0.02	-1.35	1.32	0.03	-1.29	1.36	0.02	-1.31	1.35	-0.04	-1.36	1.30
Highest ed.: O-level[b]	0.06	-0.94	1.06	0.09	-0.90	1.08	0.09	-0.90	1.11	0.05	-0.95	1.03
Highest ed.: A-level[b]	-0.64	-1.68	0.42	-0.62	-1.67	0.43	-0.62	-1.65	0.43	-0.64	-1.70	0.40
Highest ed.: Degree[b]	-1.37	-2.50	-0.25	-1.33	-2.44	-0.22	-1.33	-2.45	-0.20	-1.39	-2.50	-0.27
Individual-level random effects for SBP												
SD(mean BP)[c]	5.39	5.15	5.63	5.39	5.16	5.63	5.38	5.15	5.62	5.39	5.16	5.63
SD(BP slope)[c]	0.65	0.55	0.75	0.65	0.55	0.75	0.65	0.55	0.75	0.65	0.54	0.74
SD(log(BPV))[c]	0.44	0.33	0.54	0.44	0.33	0.55	0.42	0.30	0.53	0.42	0.29	0.53
Cor(mean BP, BP slope)[c]	-0.08	-0.21	0.03	-0.07	-0.19	0.05	-0.09	-0.21	0.03	-0.09	-0.21	0.03
Cor(mean BP, log(BPV))[c]	0.48	0.32	0.66	0.48	0.32	0.66	0.51	0.34	0.70	0.50	0.33	0.71

Cor(BP slope, log(BPV))[c]	-0.06	-0.32	0.21	-0.07	-0.34	0.19	-0.02	-0.30	0.26	-0.05	-0.34	0.23
BPV: fixed effects												
Intercept	3.11	3.00	3.22	3.11	3.00	3.22	3.12	3.00	3.23	3.13	3.02	3.23
Age (years)[d]	9.80%	4.20%	15.58%	9.84%	4.25%	15.61%	9.64%	4.01%	15.49%	10.80%	5.05%	16.77%
Female[d]	21.04%	6.72%	36.84%	21.16%	7.03%	36.76%	21.50%	7.42%	37.05%	20.06%	5.99%	35.46%
log(Weight, kg)[e]	4.80%	1.04%	8.72%	4.78%	1.05%	8.62%	4.61%	0.85%	8.48%	5.24%	1.42%	9.20%
Height (cm)[d]	0.00%	-1.06%	1.07%	-0.02%	-1.09%	1.03%	0.08%	-1.01%	1.18%	-0.24%	-1.36%	0.89%
Residual within-clinic SD for SBP	5.62	5.53	5.70	5.62	5.53	5.70	5.62	5.53	5.70	5.61	5.53	5.70
Mean for log(LVMI): fixed effects												
Intercept	3.32	3.31	3.33	3.32	3.31	3.33	3.32	3.31	3.33	3.32	3.31	3.33
Age (years)[d]	-1.98%	-4.64%	0.72%	-2.10%	-4.79%	0.63%	-2.04%	-4.72%	0.69%	-1.94%	-4.61%	0.79%
Female[d]	-5.01%	-6.76%	-3.24%	-4.98%	-6.73%	-3.20%	-5.01%	-6.76%	-3.23%	-4.95%	-6.72%	-3.17%
Weight (kg)[d]	0.70%	0.63%	0.77%	0.70%	0.63%	0.77%	0.70%	0.63%	0.77%	0.70%	0.63%	0.78%
mean BP[c,d]	0.19%	0.00%	0.39%							0.59%	0.12%	1.37%
BP slope[c,d]				2.40%	-0.51%	5.56%				3.58%	0.01%	7.92%
log(BPV)[c,e]							0.21%	-0.23%	0.69%	-0.78%	-2.54%	0.22%
Residual SD for log(LVMI)	0.19	0.18	0.20	0.19	0.18	0.20	0.19	0.18	0.20	0.19	0.18	0.19

[a] Linear spline terms, corresponding to change per year for age ≤ 12, and for age >12, respectively.

[b] Reference category for mother's highest education qualification: CSE / None.

[c] mean BP denotes the individual-level, between-clinic, random effects for the intercept (at mean age 11.3 years) in the mean function for SBP; BP slope denotes the individual-level, between-clinic, random effects for age in the mean function for SBP; log(BPV) denotes the individual-level, between-clinic, random effects on the log-scale of the within-individual variance; i.e. these are the equivalent of u_0 , u_1 and u_2 in equation 2, respectively.

[d] estimates presented as percentage change in BPV / LVMI (on natural scale) per 1-unit increase in covariate (3), calculated (using posterior samples) as (exp(coefficient) - 1) * 100.

[e] estimates presented as percentage change in BPV / LVMI (on natural scale) per 10% increase in covariate (on natural scale) (3), calculated (using posterior samples) as ((1.10^coefficient) - 1) * 100.

Web Table 7

Web Table 7: Estimates from the joint models presented in Table 3 in the main manuscript, but with original, non-transformed, regression coefficients presented for all parameter estimates.

Model	mean BP only	BP slope only	log(BPV) only	mean BP, BP slope, log(BPV) all included
N individuals	1,813	1,813	1,813	1,813

	Mean	2.5%	97.5%	Mean	2.5%	97.5%	Mean	2.5%	97.5%	Mean	2.5%	97.5%
N clinic visits	9,693			9,693			9,693			9,693		
N BP measurements	17,777			17,777			17,777			17,777		
Mean SBP: fixed effects												
Intercept	108.23	107.28	109.18	108.23	107.29	109.17	108.21	107.26	109.14	108.23	107.29	109.16
≤12 years[a]	-0.54	-0.80	-0.27	-0.49	-0.75	-0.23	-0.51	-0.77	-0.25	-0.56	-0.83	-0.30
>12 years[a]	3.88	3.54	4.23	3.94	3.60	4.28	3.92	3.58	4.26	3.83	3.49	4.18
Female	-0.25	-0.83	0.33	-0.29	-0.87	0.30	-0.27	-0.85	0.32	-0.22	-0.81	0.36
Female*≤12 years	-0.17	-0.35	0.02	-0.16	-0.34	0.03	-0.16	-0.35	0.02	-0.17	-0.36	0.01
Female*>12 years	-1.29	-1.61	-0.96	-1.32	-1.64	-1.00	-1.31	-1.63	-0.98	-1.25	-1.58	-0.92
log(Weight, kg)	16.79	15.08	18.51	16.95	15.24	18.64	16.93	15.22	18.64	16.56	14.84	18.26
Height (cm)	0.05	-0.01	0.10	0.03	-0.02	0.08	0.04	-0.01	0.09	0.06	0.00	0.11
Maternal characteristics												
Age at delivery (years)	0.01	-0.06	0.08	0.01	-0.06	0.08	0.01	-0.06	0.08	0.01	-0.06	0.09
Parity	-0.16	-0.50	0.17	-0.17	-0.51	0.17	-0.17	-0.50	0.17	-0.17	-0.50	0.17
Highest ed.: vocational[b]	-0.02	-1.35	1.32	0.03	-1.29	1.36	0.02	-1.31	1.35	-0.04	-1.36	1.30
Highest ed.: O-level[b]	0.06	-0.94	1.06	0.09	-0.90	1.08	0.09	-0.90	1.11	0.05	-0.95	1.03
Highest ed.: A-level[b]	-0.64	-1.68	0.42	-0.62	-1.67	0.43	-0.62	-1.65	0.43	-0.64	-1.70	0.40
Highest ed.: Degree[b]	-1.37	-2.50	-0.25	-1.33	-2.44	-0.22	-1.33	-2.45	-0.20	-1.39	-2.50	-0.27
Individual-level random effects for SBP												

SD(mean BP)[c]	5.39	5.15	5.63	5.39	5.16	5.63	5.38	5.15	5.62	5.39	5.16	5.63
SD(BP slope)[c]	0.65	0.55	0.75	0.65	0.55	0.75	0.65	0.55	0.75	0.65	0.54	0.74
SD(log(BPV))[c]	0.44	0.33	0.54	0.44	0.33	0.55	0.42	0.30	0.53	0.42	0.29	0.53
Cor(mean BP, BP slope)[c]	-0.08	-0.21	0.03	-0.07	-0.19	0.05	-0.09	-0.21	0.03	-0.09	-0.21	0.03
Cor(mean BP, log(BPV))[c]	0.48	0.32	0.66	0.48	0.32	0.66	0.51	0.34	0.70	0.50	0.33	0.71
Cor(BP slope, log(BPV))[c]	-0.06	-0.32	0.21	-0.07	-0.34	0.19	-0.02	-0.30	0.26	-0.05	-0.34	0.23
BPV: fixed effects												
Intercept	3.11	3.00	3.22	3.11	3.00	3.22	3.12	3.00	3.23	3.13	3.02	3.23
Age (years)	0.09	0.04	0.14	0.09	0.04	0.15	0.09	0.04	0.14	0.10	0.05	0.15
Female	0.19	0.07	0.31	0.19	0.07	0.31	0.19	0.07	0.32	0.18	0.06	0.30
log(Weight, kg)	0.49	0.11	0.88	0.49	0.11	0.87	0.47	0.09	0.85	0.53	0.15	0.92
Height (cm)	0.00	-0.01	0.01	0.00	-0.01	0.01	0.00	-0.01	0.01	0.00	-0.01	0.01
Residual within-clinic SD for SBP	5.62	5.53	5.70	5.62	5.53	5.70	5.62	5.53	5.70	5.61	5.53	5.70
Mean for log(LVMI): fixed effects												
Intercept	3.32	3.31	3.33	3.32	3.31	3.33	3.32	3.31	3.33	3.32	3.31	3.33
Age (years)	-0.02	-0.05	0.01	-0.02	-0.05	0.01	-0.02	-0.05	0.01	-0.02	-0.05	0.01
Female	-0.05	-0.07	-0.03	-0.05	-0.07	-0.03	-0.05	-0.07	-0.03	-0.05	-0.07	-0.03
Weight (kg)	0.01	0.01	0.01	0.01	0.01	0.01	0.01	0.01	0.01	0.01	0.01	0.01
mean BP[c]	0.00	0.00	0.00							0.01	0.00	0.01

BP slope[c]				0.02	-0.01	0.05				0.03	0.00	0.08
log(BPV)[c]							0.02	-0.02	0.07	-0.08	-0.27	0.02
Residual SD for log(LVMI)	0.19	0.18	0.20	0.19	0.18	0.20	0.19	0.18	0.20	0.19	0.18	0.19

[a] Linear spline terms, corresponding to change per year for age ≤ 12, and for age >12, respectively.

[b] Reference category for mother's highest education qualification: CSE / None.

[c] mean BP denotes the individual-level, between-clinic, random effects for the intercept (at mean age 11.3 years) in the mean function for SBP; BP slope denotes the individual-level, between-clinic, random effects for age in the mean function for SBP; log(BPV) denotes the individual-level, between-clinic, random effects on the log-scale of the within-individual variance; i.e. these are the equivalent of u_0 , u_1 and u_2 in equation 2, respectively.

Web Appendix 2: Examples using Stan

Contents

Introduction **23**

A note on number of repeated measures **24**

Loading libraries; choosing options **24**

2-level model with complex within-individual variability, including random effect **25**
Equation 25
Simulating data 25
Stan 27
A note on library(brms) 30

Adding a individual-level outcome: joint model **31**
Equation 31
Simulating individual-level data 31
Stan 31

Introducing an additional, lower level **36**
Equation 36
Simulating data 36
Stan 39

NB this supplementary data is from: Richard M.A. Parker, George Leckie, Harvey Goldstein, Laura D. Howe, Jon Heron, Alun D. Hughes, David M. Phillippo, Kate Tilling. "Joint modelling of individual trajectories, within-individual variability and a later outcome: systolic blood pressure through childhood and left ventricular mass in early adulthood"

Introduction

In this Web Appendix, a series of models are fitted, of increasing complexity, to simulated data, concluding with a three-level joint model with a repeatedly-measured outcome and an individual-level outcome, and with complex within-individual variability.

The R script below fits models in Stan. It uses 4 chains for each model, and otherwise the default number of total iterations and burnin/warmup iterations (and thinning) are used (see `?stan` for details). Dataset sample sizes and the number of chain iterations used can naturally be adjusted to facilitate better estimation, to run these example models more/less quickly, etc.

Stan is called via the R package `rstan`. To install `rstan`, please see https://github.com/stan-dev/rstan/wiki/RStan-Getting-Started. Stan offers an efficient method of exploring complex posterior probabilities through its use of Hamiltonian Monte Carlo (HMC) and no-U-turn samplers (NUTS).

The Stan models use a non-centered parameterisation for the random effects, fitting them as independent standard Normals (N(0, 1)). Cholesky factorisation allows the covariance matrix for the random effects to

be removed from the prior and recovered in the `transformed parameters` block. Since these optimisations change the shape of the posterior the HMC algorithm samples from, they can improve the efficiency with which multilevel models are estimated. Vectorisation (*cf.* the use of for loops, for example) also contributes to optimising the model.

(Cholesky factorisation can be used to derive the square-root of a symmetrical matrix, e.g., in R script:)

```
# symmetrical matrix, R:
R <- matrix(c(1, 0.5,
              0.5, 1), nrow = 2, ncol = 2)
# use Cholesky decomposition to derive lower triangular square root of R:
L <- t(chol(R))
# recover original symmetrical matrix:
L %*% t(L)
```

In the Stan model code below, `diag_pre_multiply(sigma_u, cholesky_corr_u)` calculates the Cholesky factor of the covariance matrix, multiplying `sigma_u` (as a diagonal matrix) with the Cholesky factor of the random effect correlation matrix. (Note `diag_pre_multiply(vector, matrix) = diag_matrix(vector) * matrix`, where `diag_matrix(vector)` would be (in R script), for example, `matrix(c(sigma_u[1], 0, 0, sigma_u[2]), nrow = 2, ncol = 2, byrow = FALSE)`).

When the resulting Cholesky factor of the random effect covariance matrix is multiplied by the independent (standard Normal) random effects, `z_u`, the correlated random effects `u` are recovered. (`z_u` is a matrix with `n_u` rows and `J` columns, where `n_u` is the number of random effects allowed to covary, and `J` denotes the number of individuals).

There are a wide variety of references providing further guidance and information, a small selection of which are listed at the end of this Web Appendix (4-8).

A note on number of repeated measures

With regard to how many repeated measures are needed to estimate these models, then it can be helpful to examine what complexity of model could be fitted if the measures were taken at the same age for all individuals. For example, in the case of three measures per person, each at exactly the same ages, then three means and six variance/covariances could be estimated from the data. Such a dataset would be compatible with a model with individual-level random effects for mean and slope, plus random error, as this model estimates two means (intercept and slope) and four variance/covariances (variance for mean and slope, covariance between them, random error). However, we could not estimate a model including random terms for mean, slope and BPV, plus random error, since this model estimates seven variance/covariances (the variation for each of mean, slope, BPV and random error (four variances), plus the three covariances between mean, slope and within-individual variability). However, we could estimate the more elaborate model in the case of four measures per person (each at exactly the same ages), as ten variance/covariances could be estimated from these data.

Loading libraries; choosing options

```
# For Stan model fits:
library("rstan")
# For execution on a local, multicore CPU with excess RAM, rstan
# recommends calling:
options(mc.cores = parallel::detectCores())
## Check how many cores detected:
# getOption("mc.cores", 1L)
```

```r
# To avoid recompilation of unchanged Stan programs, rstan
# recommends calling:
rstan_options(auto_write = TRUE)
# The following can improve execution time, but can also result in errors
# on some processors:
# Sys.setenv(LOCAL_CPPFLAGS = '-march=native')
library("shinystan") # further diagnostics
```

2-level model with complex within-individual variability, including random effect

Equation

$$y_{1ij} = \beta_0 + \beta_1 \mathrm{x}_{1ij} + u_{0j} + u_{1j}\mathrm{x}_{1ij} + e_{ij}$$

$$\begin{pmatrix} u_{0j} \\ u_{1j} \\ u_{2j} \end{pmatrix} \sim \mathrm{N}\left[\begin{pmatrix} 0 \\ 0 \\ 0 \end{pmatrix}, \begin{pmatrix} \sigma_0^2 & & \\ \sigma_{01} & \sigma_1^2 & \\ \sigma_{02} & \sigma_{12} & \sigma_2^2 \end{pmatrix}\right]$$

$$e_{ij} \sim \mathrm{N}(0, \sigma_{eij}^2), \quad \ln(\sigma_{eij}^2) = \alpha_0 + \alpha_1 \mathrm{x}_{1ij} + u_{2j}$$

Simulating data

```r
# number of individuals:
J <- 1000
# number of repeated measures per individual:
n <- 10
# individual-level indicator at level 1 (observation level):
Ind_L1 <- rep(1:J, each = n)
# total number of observations:
N <- n * J

# design matrix for fixed part of mean function for y1;
# contains constant of ones and a covariate (for simplicity, randomly-drawn
# from a standard Normal distribution):
X_y1_mu <- cbind(rep(1, times = N),
             rnorm(n = N, mean = 0, sd = 1))

# design matrix for random part of mean function for y1,
# and fixed part of within-individual variance function for y1 (all same):
X_y1_wiv <- Z_y1_mu <- X_y1_mu
# design matrix for random part of within-individual variance function
# for y1 (just a constant):
Z_y1_wiv <- as.matrix(X_y1_mu[, 1])

# number of fixed effects (betas) in mean function for y1:
n_b <- ncol(X_y1_mu)
# coefficient values for fixed effects in mean function for y1:
beta <- c(1, 1)
```

```r
# number of fixed effects (alphas) in within-individual variance function for y1:
n_a <- ncol(X_y1_wiv)
# coefficient values for fixed effects in within-individual variance function
# for y1:
alpha <- c(1, 1)

# number of individual-level random effects in mean function for y1:
n_u_mu <- ncol(Z_y1_mu)
# number of individual-level random effects in within-individual variance
# function for y1:
n_u_wiv <- ncol(Z_y1_wiv)
# number of REs in total:
n_u_total <- n_u_mu + n_u_wiv
# number of unique covariances between random effects:
n_cov <- (n_u_total^2 - n_u_total) / 2

# SDs of random effects, and their correlations:
sigma_u <- c(0.7, 0.6, 0.4)
rho_01 <- 0.5
rho_02 <- 0.3
rho_12 <- 0.4

# correlation and covariance matrices for random effects:
corr_u <- matrix(, nrow = n_u_total, ncol = n_u_total)
corr_u[lower.tri(corr_u, diag = FALSE)] <- c(rho_01, rho_02, rho_12)
diag(corr_u) <- rep(1, times = n_u_total)
corr_u[upper.tri(corr_u)] <- t(corr_u)[upper.tri(corr_u)]
cov_u <- diag(sigma_u) %*% corr_u %*% diag(sigma_u)

# generate random effects:
u <- MASS::mvrnorm(n = J,
                   mu = rep(0, times = n_u_total),
                   Sigma = cov_u)
# expand out to level 1:
u_long <- u[Ind_L1, ]

# generate fixed and random part (at level 2) of model for mean of y1:
fixpart_y1_mu <- as.vector(X_y1_mu %*% beta)
randpart_y1_mu <- rowSums(Z_y1_mu * u_long[, 1:n_u_mu])

# generate fixed and random part (at level 2) of model for within-individual
# variance of y1:
fixpart_y1_wiv <- as.vector(X_y1_wiv %*% alpha)
randpart_y1_wiv <- rowSums(Z_y1_wiv * u_long[, (n_u_mu + 1):n_u_total])

# generate level 1 residuals:
log_sigma2_e <- fixpart_y1_wiv + randpart_y1_wiv
sigma_e <- sqrt(exp(log_sigma2_e))
e <- rnorm(n = N, mean = 0, sd = sigma_e)

## generate repeatedly-measured outcome
y1 <- fixpart_y1_mu + randpart_y1_mu + e
```

Stan

Specifying priors

To be adjusted as appropriate.

In this particular example, for the fixed effects in the mean function for y1, we use the equivalent as had the variables been standardised - of Normal(mean(y1), SD = 10) for intercept and Normal(0, SD = 2.5) for covariates (9).

For the coefficients in the function for the within-individual variance, we also use Normal priors. For the location (mean) of the prior for the coefficient of the intercept we use an estimate of the within-individual SD gleaned from a simpler (random slopes) model, with zero for the location (mean) of the prior for the coefficient of the covariate. With regard to the scale of these priors, in this example we have chosen a value which would predict sigma_e of between c. 0.1 to 42 (i.e. very weakly informative, in this context) with +/- 2 SDs of a standard Normal predictor.

```
# priors (adjust as appropriate):
# fixed effects:
beta_prior_loc <- c(mean(y1), rep(0, times = n_b - 1))
beta_prior_scale_denom <- sd(X_y1_mu[, 2])
beta_prior_scale <- (2.5 * sd(y1)) / beta_prior_scale_denom
beta_prior_scale <- c(10 * sd(y1), beta_prior_scale)

within_ind_SD_estimate <- 2.1
alpha_prior_loc <- log(within_ind_SD_estimate^2)
alpha_prior_scale <- prior_sd <- 3
sqrt(exp(alpha_prior_loc - (2 * prior_sd)))
# [1] 0.1045528
sqrt(exp(alpha_prior_loc + (2 * prior_sd)))
# [1] 42.17963
alpha_prior_scale_denom <- sd(X_y1_wiv[, 2])
rescaled_prior_sd <- prior_sd / alpha_prior_scale_denom
rescaled_prior_sd
alpha_prior_scale <- c(alpha_prior_scale, rescaled_prior_sd)
alpha_prior_loc <- c(alpha_prior_loc, rep(0, times = n_a - 1))

# random effects:
# LKJ prior for correlation matrix:
LKJcorr_prior <- 2
# half-Cauchy for SDs:
sigma_u_prior_loc <- 0
sigma_u_prior_scale <- 10
```

Creating the data object

```
data <- list(N = N,
             J = J,
             n_b = n_b,
             n_a = n_a,
             n_u_mu = n_u_mu,
             n_u_total = n_u_total,
             n_cov = n_cov,
             X_y1_mu = X_y1_mu,
```

```r
             Z_y1_mu = Z_y1_mu,
             X_y1_wiv = X_y1_wiv,
             Z_y1_wiv = Z_y1_wiv,
             y1 = y1,
             Ind_L1 = Ind_L1,
             beta_prior_loc = beta_prior_loc,
             beta_prior_scale = beta_prior_scale,
             alpha_prior_loc = alpha_prior_loc,
             alpha_prior_scale = alpha_prior_scale,
             LKJcorr_prior = LKJcorr_prior,
             sigma_u_prior_loc = sigma_u_prior_loc,
             sigma_u_prior_scale = sigma_u_prior_scale)
```

Specifying the model

NB: Assuming this Stan program is saved in its own file.

```r
writeLines(readLines("wiv_RE.stan"))
```

```
## //This Stan model fits a 2-level multilevel (Gaussian) model, with complex
## //level 1 variability, including random effects in the level 1 variability
## //function. It assumes that the subject indicator (Ind_L1) is numbered in
## //ascending order from 1, in steps of 1. It also assumes that any rows with
## //missing observations have been dropped. It uses user-specified priors.
## data {
##   //num observations (level 1)
##   int<lower=0> N;
##   //num subjects (level 2)
##   int<lower=0> J;
##   //num FEs (betas) in mean fun. for y1
##   int<lower=1> n_b;
##   //num FEs (alphas) in wiv fun. for y1
##   int<lower=1> n_a;
##   //num REs in mean fun. for y1
##   int<lower=1> n_u_mu;
##   //total num REs for y1
##   int<lower=1> n_u_total;
##   //num unique covariances between REs
##   int<lower=1> n_cov;
##   //design matrix:fixed part mean fun. for y1
##   matrix[N, n_b] X_y1_mu;
##   //design matrix:random part mean fun. for y1
##   matrix[N, n_u_mu] Z_y1_mu;
##   //design matrix:fixed part wiv fun. for y1
##   matrix[N, n_a] X_y1_wiv;
##   //design matrix:random part wiv fun. for y1
##   matrix[N, n_u_total - n_u_mu] Z_y1_wiv;
##   //repeatedly-measured outcome
##   vector[N] y1;
##   //subject indicator (of N-length)
##   int<lower=1,upper=J> Ind_L1[N];
##   //location and scale of priors for betas
##   vector[n_b] beta_prior_loc;
```

```
##   vector<lower=0>[n_b] beta_prior_scale;
##   //location and scale of priors for alphas
##   vector[n_a] alpha_prior_loc;
##   vector<lower=0>[n_a] alpha_prior_scale;
##   //eta for LKJcorr prior for RE correlations
##   real<lower=0> LKJcorr_prior;
##   //location and scale of prior for RE SDs
##   real sigma_u_prior_loc;
##   real<lower=0> sigma_u_prior_scale;
## }
## parameters {
##   //FE coefficients in mean function for y_repeat
##   vector[n_b] beta;
##   //FE coefficients in within-individual variance function for y_repeat
##   vector[n_a] alpha;
##   //Cholesky factor of random effect corr matrix
##   //(i.e. corr_u = cholesky_corr_u * cholesky_corr_u')
##   cholesky_factor_corr[n_u_total] cholesky_corr_u;
##   //random effect SDs (lower bound ensures half-Cauchy)
##   vector<lower=0>[n_u_total] sigma_u;
##   //unscaled random effects (N(0,1))
##   matrix[n_u_total, J] z_u;
## }
## transformed parameters {
##   //scaled random effects
##   matrix[J, n_u_total] u;
##   u = (diag_pre_multiply(sigma_u, cholesky_corr_u) * z_u)';
## }
## model {
##   //priors
##   beta ~ normal(beta_prior_loc, beta_prior_scale);
##   alpha ~ normal(alpha_prior_loc, alpha_prior_scale);
##   //normal() not applicable to matrices, hence to_vector
##   //(treats it like a vector but maintains matrix data type)
##   to_vector(z_u) ~ normal(0, 1);
##   cholesky_corr_u ~ lkj_corr_cholesky(LKJcorr_prior);
##   sigma_u ~ cauchy(sigma_u_prior_loc, sigma_u_prior_scale);
##   //likelihood; uses vectorisation and matrix multiplication
##   //NB: if want to model log(sigma) instead, then remove sqrt()
##   //from within-individual variability function
##   y1 ~ normal(X_y1_mu * beta
##               + rows_dot_product(Z_y1_mu, u[Ind_L1, 1:n_u_mu]),
##               sqrt(exp(X_y1_wiv * alpha
##               + rows_dot_product(Z_y1_wiv, u[Ind_L1, (n_u_mu + 1):n_u_total]))));
## }
## generated quantities {
##   corr_matrix[n_u_total] corr_u_complete;
##   vector<lower=-1, upper=1>[n_cov] corr_u;
##   //return correlation matrix (dropping redundant elements)
##   //adapted from brms model code
##   corr_u_complete = multiply_lower_tri_self_transpose(cholesky_corr_u);
##   for (k in 1:n_u_total) {
##     for (j in 1:(k - 1)) {
##       corr_u[choose(k - 1, 2) + j] = corr_u_complete[j, k];
```

```
##     }
##   }
## }
```

Fitting the model

```
params_for_summary <- c("beta",
                        "alpha",
                        "sigma_u",
                        "corr_u")
chains <- 4

# (include u (etc.) in pars if wish to save e.g. residuals at that level)
stan_fit <- stan(file = "wiv_RE.stan",
                 data = data,
                 pars = params_for_summary,
                 chains = chains)
```

Inspecting results

```
print(stan_fit, pars = params_for_summary)

# Launching shinystan to check diagnostics
stan_fit_shiny <- as.shinystan(stan_fit, pars = params_for_summary)
launch_shinystan(stan_fit_shiny)
```

A note on library(brms)

Note that a 2-level model with complex within-individual variability, including a random effect, can also be fitted via the `brms` package (10, 11) in R.

For instance, the following example would fit a random intercept and slope (for covariate `x1`) for the mean of repeatedly-measured outcome `y1`, as well as allowing the within-individual SD (*cf.* variance) to depend on the covariate and a random effect. Here the `s` (although could be any value, as long as same in each set of parentheses) indicates that the random effects are to be modelled as correlated.

Whilst such a model can be succinctly fitted in `brms`, it is not currently possible to fit joint multilevel models in which the outcomes are at different levels and random effects are fitted as predictors, via the package.

```
library("brms")
brms_fit <- brm(bf(y1 ~ 1 + x1 + (1 + x1 |s| Ind_L1),
                   sigma ~ 1 + x1 + (1 |s| Ind_L1)),
             data = data,
             family = gaussian()
)
```

Adding a individual-level outcome: joint model

Equation

$$y_{1ij} = \beta_0 + \beta_1 \mathrm{x}_{1ij} + u_{0j} + u_{1j}\mathrm{x}_{1ij} + e_{ij}$$

$$y_{2j} = \gamma_0 + \gamma_1 \mathrm{x}_{2j} + \gamma_2 u_{0j} + \gamma_3 u_{1j} + \gamma_4 u_{2j} + u_{3j}$$

$$\begin{pmatrix} u_{0j} \\ u_{1j} \\ u_{2j} \\ u_{3j} \end{pmatrix} \sim \mathrm{N}\left[\begin{pmatrix} 0 \\ 0 \\ 0 \\ 0 \end{pmatrix}, \begin{pmatrix} \sigma_0^2 & & & \\ \sigma_{01} & \sigma_1^2 & & \\ \sigma_{02} & \sigma_{12} & \sigma_2^2 & \\ 0 & 0 & 0 & \sigma_3^2 \end{pmatrix}\right]$$

$$e_{ij} \sim \mathrm{N}(0, \sigma^2_{eij}), \quad \ln(\sigma^2_{eij}) = \alpha_0 + \alpha_1 \mathrm{x}_{1ij} + u_{2j}$$

Simulating individual-level data

(Assuming using the occasion-level data simulated earlier.)

```
# design matrix for observed covariates in mean function for y2:
X_y2_obs <- cbind(rep(1, times = J),
                  rnorm(n = J, mean = 0, sd = 1))
# number of fixed effects (gammas) for observed covariates
# in mean function for y2:
n_g_obs <- ncol(X_y2_obs)
# number of fixed effects (gammas) for REs fitted as exposures
# in mean function for y2:
n_g_RE <- ncol(u)
# design matrix for mean function for y2, incl. REs as exposures:
X_y2 <- cbind(X_y2_obs, u)
# total number of exposures in mean function for y2:
n_g_total <- n_g_obs + n_g_RE

# coefficient values for fixed effects in mean function for y2:
gamma <- c(0, 1.2, 0.2, 0.02, 0.4)

# residual SD for individual-level outcome, y2
sigma_y2 <- 0.5

# generate fixed and random part of model for mean of y2:
fixpart_y2 <- as.vector(X_y2 %*% gamma)
randpart_y2 <- rnorm(n = J, mean = 0, sd = sigma_y2)

# generate individual-level outcome:
y2 <- fixpart_y2 + randpart_y2
```

Stan

Specifying priors

To be adjusted as appropriate (and again assuming using the occasion-level data simulated earlier).

In this particular example, for the fixed effects in the mean function for y1, we use the equivalent as had the variables been standardised - of Normal(mean(y1), SD = 10) for intercept and Normal(0, SD = 2.5) for covariates (9). We do the same for the coefficients in the equation for the individual-level outcome, y2, as well - we use the random effects from the dataset we simulated to get an idea of their SDs, but can of course use estimates of the SD of the random effects from simpler (e.g. univariate outcome) models fitted to actual data for same purpose.

For the coefficients in the function for the within-individual variance, we also use Normal priors. For the location (mean) of the prior for the coefficient of the intercept we use an estimate of the within-individual SD gleaned from a simpler (random slopes) model, with zero for the location (mean) of the prior for the coefficient of the covariate. With regard to the scale of these priors, in this example we have chosen a value which would predict sigma_e of between c. 0.1 to 42 (i.e. very weakly informative, in this context) with +/- 2 SDs of a standard Normal predictor.

```
# priors (adjust as appropriate):
# fixed effects:
beta_prior_loc <- c(mean(y1), rep(0, times = n_b - 1))
beta_prior_scale_denom <- sd(X_y1_mu[, 2])
beta_prior_scale <- (2.5 * sd(y1)) / beta_prior_scale_denom
beta_prior_scale<- c(10 * sd(y1), beta_prior_scale)

within_ind_SD_estimate <- 2.1
alpha_prior_loc <- log(within_ind_SD_estimate^2)
alpha_prior_scale <- prior_sd <- 3
sqrt(exp(alpha_prior_loc - (2 * prior_sd)))
# [1] 0.1045528
sqrt(exp(alpha_prior_loc + (2 * prior_sd)))
# [1] 42.17963
alpha_prior_scale_denom <- sd(X_y1_wiv[, 2])
rescaled_prior_sd <- prior_sd / alpha_prior_scale_denom
rescaled_prior_sd
alpha_prior_scale <- c(alpha_prior_scale, rescaled_prior_sd)
alpha_prior_loc <- c(alpha_prior_loc, rep(0, times = n_a - 1))

# random effects:
# LKJ prior for correlation matrix:
LKJcorr_prior <- 2
# half-Cauchy for SDs:
sigma_u_prior_loc <- 0
sigma_u_prior_scale <- 10
sigma_y2_prior_loc <- 0
sigma_y2_prior_scale <- 10

gamma_obs_prior_loc <- c(mean(y2), rep(0, times = n_g_obs - 1))
gamma_obs_prior_scale_denom <- sd(X_y2_obs[, n_g_obs])
gamma_obs_prior_scale <- (2.5 * sd(y2)) / gamma_obs_prior_scale_denom
gamma_obs_prior_scale <- c(10 * sd(y2), gamma_obs_prior_scale)

gamma_RE_prior_scale_denom <- apply(u, MARGIN = 2, sd)
gamma_RE_prior_scale <- (2.5 * sd(y2)) / gamma_RE_prior_scale_denom
gamma_RE_prior_loc <- rep(0, times = n_g_RE)
```

Creating the data object

```r
data <- list(N = N,
             J = J,
             n_b = n_b,
             n_a = n_a,
             n_g_obs = n_g_obs,
             n_g_RE = n_g_RE,
             n_u_mu = n_u_mu,
             n_u_total = n_u_total,
             n_cov = n_cov,
             X_y1_mu = X_y1_mu,
             Z_y1_mu = Z_y1_mu,
             X_y1_wiv = X_y1_wiv,
             Z_y1_wiv = Z_y1_wiv,
             X_y2_obs = X_y2_obs,
             y1 = y1,
             y2 = y2,
             Ind_L1 = Ind_L1,
             beta_prior_loc = beta_prior_loc,
             beta_prior_scale = beta_prior_scale,
             alpha_prior_loc = alpha_prior_loc,
             alpha_prior_scale = alpha_prior_scale,
             gamma_obs_prior_loc = gamma_obs_prior_loc,
             gamma_obs_prior_scale = gamma_obs_prior_scale,
             gamma_RE_prior_loc = gamma_RE_prior_loc,
             gamma_RE_prior_scale = gamma_RE_prior_scale,
             LKJcorr_prior = LKJcorr_prior,
             sigma_u_prior_loc = sigma_u_prior_loc,
             sigma_u_prior_scale = sigma_u_prior_scale,
             sigma_y2_prior_loc = sigma_y2_prior_loc,
             sigma_y2_prior_scale = sigma_y2_prior_scale)
```

Specifying the model

NB: Assuming this Stan program is saved in its own file.

```r
writeLines(readLines("joint_L2.stan"))
```

```
## //This Stan model fits a 2-level joint (bivariate Gaussian outcome) model,
## //with one of the outcomes at level 1 (occasion-level), and the other at
## //level 2 (individual-level). As well as observed covariates, it fits random
## //effects for the mean of the occasion-level (level 1) outcome, and also
## //random effects for within-individual variability in that outcome. These
## //random effects are also fitted as predictors of the subject-level outcome,
## //within the same joint model. It assumes that the subject indicator (Ind_L1)
## //is numbered in ascending order from 1, in steps of 1, and that these values
## //correspond to the row order of the individual-level variables
## //(y2; X_y2_obs). It also assumes that any rows with missing observations
## //have been dropped. It uses user-specified priors.
## data {
##   //num observations (level 1)
##   int<lower=0> N;
```

```
##   //num subjects (level 2)
##   int<lower=0> J;
##   //num FEs (betas) in mean fun. for y1
##   int<lower=1> n_b;
##   //num FEs (alphas) in wiv fun. for y1
##   int<lower=1> n_a;
##   //num observed exposures for y2
##   int<lower=1> n_g_obs;
##   //num REs fitted as exposures for y2
##   int<lower=1> n_g_RE;
##   //num REs in mean fun. for y1
##   int<lower=1> n_u_mu;
##   //total num REs for y1
##   int<lower=1> n_u_total;
##   //num unique covariances between REs for y1
##   int<lower=1> n_cov;
##   //design matrix:fixed part mean fun. for y1
##   matrix[N, n_b] X_y1_mu;
##   //design matrix:random part mean fun. for y1
##   matrix[N, n_u_mu] Z_y1_mu;
##   //design matrix:fixed part wiv fun. for y1
##   matrix[N, n_a] X_y1_wiv;
##   //design matrix:random part wiv fun. for y1
##   matrix[N, n_u_total - n_u_mu] Z_y1_wiv;
##   //design matrix:observed exposures for y2
##   matrix[J, n_g_obs] X_y2_obs;
##   //repeatedly-measured outcome
##   vector[N] y1;
##   //individual-level outcome
##   vector[J] y2;
##   //subject indicator (of N-length)
##   int<lower=1,upper=J> Ind_L1[N];
##   //location and scale of priors for betas
##   vector[n_b] beta_prior_loc;
##   vector<lower=0>[n_b] beta_prior_scale;
##   //location and scale of priors for alphas
##   vector[n_a] alpha_prior_loc;
##   vector<lower=0>[n_a] alpha_prior_scale;
##   //location and scale of priors for observed exposures for y2
##   vector[n_g_obs] gamma_obs_prior_loc;
##   vector<lower=0>[n_g_obs] gamma_obs_prior_scale;
##   //location and scale of priors for RE exposures for y2
##   vector[n_g_RE] gamma_RE_prior_loc;
##   vector<lower=0>[n_g_RE] gamma_RE_prior_scale;
##   //eta for LKJcorr prior for RE correlations
##   real<lower=0> LKJcorr_prior;
##   //location and scale of priors for RE SDs
##   real sigma_u_prior_loc;
##   real<lower=0> sigma_u_prior_scale;
##   //location and scale of prior for residual SD for y2
##   real sigma_y2_prior_loc;
##   real<lower=0> sigma_y2_prior_scale;
## }
## parameters {
```

```
##   //FE coefficients in mean function for y1
##   vector[n_b] beta;
##   //FE coefficients in within-individual variance function for y1
##   vector[n_a] alpha;
##   //FE coefficients of observed exposures for y2
##   vector[n_g_obs] gamma_obs;
##   //FE coefficients of RE exposures for y2
##   vector[n_g_RE] gamma_RE;
##   //Cholesky factor of random effect corr matrix
##   //(i.e. corr_u = cholesky_corr_u * cholesky_corr_u')
##   cholesky_factor_corr[n_u_total] cholesky_corr_u;
##   //random effect SDs (lower bound ensures half-Cauchy)
##   vector<lower=0>[n_u_total] sigma_u;
##   //unscaled random effects (N(0,1))
##   matrix[n_u_total, J] z_u;
##   //residual SD for y2 (lower bound ensures half-Cauchy)
##   real<lower=0> sigma_y2;
## }
## transformed parameters {
##   //scaled random effects
##   matrix[J, n_u_total] u;
##   u = (diag_pre_multiply(sigma_u, cholesky_corr_u) * z_u)';
## }
## model {
##   //priors
##   beta ~ normal(beta_prior_loc, beta_prior_scale);
##   alpha ~ normal(alpha_prior_loc, alpha_prior_scale);
##   gamma_obs ~ normal(gamma_obs_prior_loc, gamma_obs_prior_scale);
##   gamma_RE ~ normal(gamma_RE_prior_loc, gamma_RE_prior_scale);
##   //normal() not applicable to matrices, hence to_vector
##   //(treats it like a vector but maintains matrix data type)
##   to_vector(z_u) ~ normal(0, 1);
##   cholesky_corr_u ~ lkj_corr_cholesky(LKJcorr_prior);
##   sigma_u ~ cauchy(sigma_u_prior_loc, sigma_u_prior_scale);
##   sigma_y2 ~ cauchy(sigma_y2_prior_loc, sigma_y2_prior_scale);
##   //likelihood; uses vectorisation and matrix multiplication
##   //NB: if want to model log(sigma) instead, then remove sqrt()
##   //from within-individual variability function
##   y1 ~ normal(X_y1_mu * beta
##               + rows_dot_product(Z_y1_mu, u[Ind_L1, 1:n_u_mu]),
##               sqrt(exp(X_y1_wiv * alpha
##               + rows_dot_product(Z_y1_wiv, u[Ind_L1, (n_u_mu + 1):n_u_total]))));
##   y2 ~ normal(X_y2_obs * gamma_obs + u * gamma_RE, sigma_y2);
## }
## generated quantities {
##   corr_matrix[n_u_total] corr_u_complete;
##   vector<lower=-1, upper=1>[n_cov] corr_u;
##   //return correlation matrix (dropping redundant elements)
##   //adapted from brms model code
##   corr_u_complete = multiply_lower_tri_self_transpose(cholesky_corr_u);
##   for (k in 1:n_u_total) {
##     for (j in 1:(k - 1)) {
##       corr_u[choose(k - 1, 2) + j] = corr_u_complete[j, k];
##     }
```

```
##   }
## }
```

Fitting the model

```
params_for_summary <- c("beta",
                        "alpha",
                        "gamma_obs",
                        "gamma_RE",
                        "sigma_y2",
                        "sigma_u",
                        "corr_u")
chains <- 4

# (include u (etc.) in pars if wish to save e.g. residuals at that level)
stan_fit <- stan(file = "joint_L2.stan",
                 data = data,
                 pars = params_for_summary,
                 chains = chains)
```

Inspecting results

```
print(stan_fit, pars = params_for_summary)

# Launching shinystan to check diagnostics
stan_fit_shiny <- as.shinystan(stan_fit, pars = params_for_summary)
launch_shinystan(stan_fit_shiny)
```

Introducing an additional, lower level

Equation

$$y_{1hij} = \beta_0 + \beta_1 \mathrm{x}_{1ij} + u_{0j} + u_{1j}\mathrm{x}_{1ij} + e_{ij} + \epsilon_{hij}$$

$$y_{2j} = \gamma_0 + \gamma_1 \mathrm{x}_{2j} + \gamma_2 u_{0j} + \gamma_3 u_{1j} + \gamma_4 u_{2j} + u_{3j}$$

$$\begin{pmatrix} u_{0j} \\ u_{1j} \\ u_{2j} \\ u_{3j} \end{pmatrix} \sim \mathrm{N}\left[\begin{pmatrix} 0 \\ 0 \\ 0 \\ 0 \end{pmatrix}, \begin{pmatrix} \sigma^2_{u0} & & & \\ \sigma_{u01} & \sigma^2_{u1} & & \\ \sigma_{u02} & \sigma_{u12} & \sigma^2_{u2} & \\ 0 & 0 & 0 & \sigma^2_{u3} \end{pmatrix}\right]$$

$$e_{ij} \sim \mathrm{N}(0, \sigma^2_{eij}), \quad \ln(\sigma^2_{eij}) = \alpha_0 + \alpha_1 \mathrm{x}_{1ij} + u_{2j}$$

$$\epsilon_{hij} \sim \mathrm{N}(0, \sigma^2_{\epsilon})$$

Simulating data

```r
# number of individuals:
J <- 1000
# number of clincs attended per individual:
num_clinics_per_J <- 10
# number of measurements taken per clinic
n <- 2
# total number of clinic sessions attended:
total_num_clinics <- num_clinics_per_J * J
# total number of observations:
N <- n * num_clinics_per_J * J

# individual-level indicator at level 2 (clinic level):
Ind_L2 <- rep(1:J, each = num_clinics_per_J)
# individual-level indicator at level 1 (observation level):
Ind_L1 <- rep(1:J, each = n * num_clinics_per_J)
# clinic_level indicator at level 1 (observation level)
clinic_L1 <- rep(1:total_num_clinics, each = n)

# design matrix at level 2 (clinic level) for fixed part of mean function for
# y1; contains constant of ones and a covariate (for simplicity, randomly-drawn
# from a standard Normal distribution):
X_y1_mu_L2 <- cbind(rep(1, times = total_num_clinics),
                    rnorm(n = total_num_clinics, mean = 0, sd = 1))

# design matrix at level 2 (clinic level) for random part of mean function for
# y1 and fixed part of within-individual variance function for y1 (all same):
X_y1_wiv_L2 <- Z_y1_mu_L2 <- X_y1_mu_L2
# design matrix at level 2 (clinic level) for random part of within-individual
# variance function for y1 (just constant):
Z_y1_wiv_L2 <- as.matrix(X_y1_mu_L2[, 1])

# expanding out to create design matrices at level 1 (observation level):
X_y1_mu_L1 <- cbind(rep(1, times = N),
                   rep(X_y1_mu_L2[, 2], each = n))
X_y1_wiv_L1 <- Z_y1_mu_L1 <- X_y1_mu_L1
Z_y1_wiv_L1 <- as.matrix(X_y1_mu_L1[, 1])

# number of fixed effects (betas) in mean function for y1:
n_b <- ncol(X_y1_mu_L2)
# coefficient values for fixed effects in mean function for y1:
beta <- c(1, 1)

# number of fixed effects (alphas) in within-individual variance function for y1:
n_a <- ncol(X_y1_wiv_L2)
# coefficient values for fixed effects in within-individual variance function
# for y1:
alpha <- c(1, 1)

# number of individual-level random effects in mean function for y1:
n_u_mu <- ncol(Z_y1_mu_L2)
# number of individual-level random effects in within-individual variance
# function for y1:
n_u_wiv <- ncol(Z_y1_wiv_L2)
```

```r
# number of REs in total:
n_u_total <- n_u_mu + n_u_wiv
# number of unique covariances between random effects:
n_cov <- (n_u_total^2 - n_u_total) / 2

# SDs of random effects, and their correlations:
sigma_u <- c(0.7, 0.6, 0.4)
rho_01 <- 0.5
rho_02 <- 0.3
rho_12 <- 0.4

# correlation and covariance matrices for random effects:
corr_u <- matrix(, nrow = n_u_total, ncol = n_u_total)
corr_u[lower.tri(corr_u, diag = FALSE)] <- c(rho_01, rho_02, rho_12)
diag(corr_u) <- rep(1, times = n_u_total)
corr_u[upper.tri(corr_u)] <- t(corr_u)[upper.tri(corr_u)]
cov_u <- diag(sigma_u) %*% corr_u %*% diag(sigma_u)

# generate random effects:
u <- MASS::mvrnorm(n = J,
                   mu = rep(0, times = n_u_total),
                   Sigma = cov_u)
# expand out to level 2:
u_long <- u[Ind_L2, ]

# generate fixed and random part (at level 2) of model for mean of y1:
fixpart_y1_mu <- as.vector(X_y1_mu_L2 %*% beta)
randpart_y1_mu <- rowSums(Z_y1_mu_L2 * u_long[, 1:n_u_mu])

# generate fixed and random part (at level 2) of model for within-individual
# variance of y1:
fixpart_y1_wiv <- as.vector(X_y1_wiv_L2 %*% alpha)
randpart_y1_wiv <- rowSums(Z_y1_wiv_L2 * u_long[, (n_u_mu + 1):n_u_total])

# generate random effects at the clinic level
log_sigma2_e <- fixpart_y1_wiv + randpart_y1_wiv
sigma_e <- sqrt(exp(log_sigma2_e))
e <- rnorm(n = total_num_clinics, mean = 0, sd = sigma_e)

# generate preliminary repeatedly-measured outcome at level 2 (clinic level):
y1_L2 <- fixpart_y1_mu + randpart_y1_mu + e

# expand out to 1 (observation level), adding variation as do so:
y1_L2_expanded <- rep(y1_L2, each = n)
sigma_epsilon <- 0.5
y1 <- rnorm(n = length(y1_L2_expanded), mean = y1_L2_expanded, sd = sigma_epsilon)

# design matrix for observed covariates in mean function for y2:
X_y2_obs <- cbind(rep(1, times = J),
                  rnorm(n = J, mean = 0, sd = 1))
# number of fixed effects (gammas) for observed covariates
# in mean function for y2:
n_g_obs <- ncol(X_y2_obs)
```

```r
# number of fixed effects (gammas) for REs fitted as exposures
# in mean function for y2:
n_g_RE <- ncol(u)
# design matrix for mean function for y2, incl. REs as exposures:
X_y2 <- cbind(X_y2_obs, u)
# total number of exposures in mean function for y2:
n_g_total <- n_g_obs + n_g_RE

# coefficient values for fixed effects in mean function for y2:
gamma <- c(0, 1.2, 0.2, 0.02, 0.4)

# residual SD for individual-level outcome, y2
sigma_y2 <- 0.5

# generate fixed and random part of model for mean of y2:
fixpart_y2 <- as.vector(X_y2 %*% gamma)
randpart_y2 <- rnorm(n = J, mean = 0, sd = sigma_y2)

# generate individual-level outcome:
y2 <- fixpart_y2 + randpart_y2
```

Stan

Specifying priors

To be adjusted as appropriate.

In this particular example, for the fixed effects in the mean function for y1, we use the equivalent as had the variables been standardised - of Normal(mean(y1), SD = 10) for intercept and Normal(0, SD = 2.5) for covariates (9). We do the same for the coefficients in the equation for the individual-level outcome, y2, as well - we use the random effects from the dataset we simulated to get an idea of their SDs, but can of course use random effects from simpler models fitted to actual data for same purpose.

For the coefficients in the function for the within-individual variance, we also use Normal priors. In this example with simulated data we use the known residual SD for the location (mean) of the prior for the coefficient of the intercept, but with real data an estimate of the residual SD could be taken from a simpler model (e.g. random slope), for example, for the same purpose. Otherwise, zero is used for the location (mean) of the prior for the coefficient of the covariate. With regard to the scale of these priors, in this example we have chosen a value which would predict sigma_e of between c. 0.1 to 45 (i.e. very weakly informative, in this context) with +/- 2 SDs of a standard Normal predictor.

```r
# priors (adjust as appropriate):
# fixed effects:
beta_prior_loc <- c(mean(y1), rep(0, times = n_b - 1))
beta_prior_scale_denom <- sd(X_y1_mu_L1[, 2])
beta_prior_scale <- (2.5 * sd(y1)) / beta_prior_scale_denom
beta_prior_scale<- c(10 * sd(y1), beta_prior_scale)

within_ind_SD_estimate <- 2.1
alpha_prior_loc <- log(within_ind_SD_estimate^2)
alpha_prior_scale <- prior_sd <- 3
sqrt(exp(alpha_prior_loc - (2 * prior_sd)))
# [1] 0.1045528
sqrt(exp(alpha_prior_loc + (2 * prior_sd)))
```

```r
# [1] 42.17963
alpha_prior_scale_denom <- sd(X_y1_wiv_L2[, 2])
rescaled_prior_sd <- prior_sd / alpha_prior_scale_denom
rescaled_prior_sd
alpha_prior_scale <- c(alpha_prior_scale, rescaled_prior_sd)
alpha_prior_loc <- c(alpha_prior_loc, rep(0, times = n_a - 1))

# random effects:
# LKJ prior for correlation matrix:
LKJcorr_prior <- 2
# half-Cauchy for SDs:
sigma_u_prior_loc <- 0
sigma_u_prior_scale <- 10
sigma_y2_prior_loc <- 0
sigma_y2_prior_scale <- 10
sigma_epsilon_prior_loc <- 0
sigma_epsilon_prior_scale <- 10

gamma_obs_prior_loc <- c(mean(y2), rep(0, times = n_g_obs - 1))
gamma_obs_prior_scale_denom <- sd(X_y2_obs[, n_g_obs])
gamma_obs_prior_scale <- (2.5 * sd(y2)) / gamma_obs_prior_scale_denom
gamma_obs_prior_scale <- c(10 * sd(y2), gamma_obs_prior_scale)

gamma_RE_prior_scale_denom <- apply(u, MARGIN = 2, sd)
gamma_RE_prior_scale <- (2.5 * sd(y2)) / gamma_RE_prior_scale_denom
gamma_RE_prior_loc <- rep(0, times = n_g_RE)
```

Creating the data object

```r
data = list(N = N,
            total_num_clinics = total_num_clinics,
            J = J,
            n_b = n_b,
            n_a = n_a,
            n_g_obs = n_g_obs,
            n_g_RE = n_g_RE,
            n_u_mu = n_u_mu,
            n_u_total = n_u_total,
            n_cov = n_cov,
            X_y1_mu_L1 = X_y1_mu_L1,
            Z_y1_mu_L1 = Z_y1_mu_L1,
            X_y1_wiv_L2 = X_y1_wiv_L2,
            Z_y1_wiv_L2 = Z_y1_wiv_L2,
            X_y2_obs = X_y2_obs,
            y1 = y1,
            y2 = y2,
            Ind_L1 = Ind_L1,
            Ind_L2 = Ind_L2,
            clinic_L1 = clinic_L1,
            beta_prior_loc = beta_prior_loc,
            beta_prior_scale = beta_prior_scale,
            alpha_prior_loc = alpha_prior_loc,
```

```r
            alpha_prior_scale = alpha_prior_scale,
            gamma_obs_prior_loc = gamma_obs_prior_loc,
            gamma_obs_prior_scale = gamma_obs_prior_scale,
            gamma_RE_prior_loc = gamma_RE_prior_loc,
            gamma_RE_prior_scale = gamma_RE_prior_scale,
            LKJcorr_prior = LKJcorr_prior,
            sigma_u_prior_loc = sigma_u_prior_loc,
            sigma_u_prior_scale = sigma_u_prior_scale,
            sigma_y2_prior_loc = sigma_y2_prior_loc,
            sigma_y2_prior_scale = sigma_y2_prior_scale,
            sigma_epsilon_prior_loc = sigma_epsilon_prior_loc,
            sigma_epsilon_prior_scale = sigma_epsilon_prior_scale)
```

Specifying the model

NB: Assuming this Stan program is saved in its own file.

```r
writeLines(readLines("joint_L3.stan"))
```

```
## //This Stan model fits a 3-level joint (bivariate Gaussian outcome) model,
## //with one of the outcomes at level 1 (occasion-level), and the other at
## //level 3 (e.g. individual-level). Here level 2 is assumed to be a clinic,
## //for example. As well as observed covariates, it fits (individual-level)
## //random effects for the mean of the occasion-level (level 1) outcome, and
## //also random effects for within-individual (between-clinic) variability in
## //that outcome. These random effects are also fitted as predictors of the
## //subject-level outcome, within the same joint model. The between-occasion
## //(within-clinic) variance is assumed constant, and not related to the
## //individual-level outcome. It assumes that the indicator variables are
## //numbered in ascending order from 1, in steps of 1, and that the values
## //denoting different individuals correspond to the row order of the
## //individual-level variables. It also assumes that any rows with missing
## //observations have been dropped. It uses user-specified priors.
## data {
##   //num observations (level 1)
##   int<lower=0> N;
##   //num clinic sessions attended (level 2)
##   int<lower=0> total_num_clinics;
##   //num subjects (level 3)
##   int<lower=0> J;
##   //num FEs (betas) in mean fun. for y1
##   int<lower=1> n_b;
##   //num FEs (alphas) in wiv fun. for y1
##   int<lower=1> n_a;
##   //num observed exposures for y2
##   int<lower=1> n_g_obs;
##   //num REs fitted as exposures for y2
##   int<lower=1> n_g_RE;
##   //num REs in mean fun. for y1
##   int<lower=1> n_u_mu;
##   //total num REs for y1
##   int<lower=1> n_u_total;
##   //num unique covariances between REs
```

```
##   int<lower=1> n_cov;
##   //design matrix (at level 1):fixed part mean fun. for y1
##   matrix[N, n_b] X_y1_mu_L1;
##   //design matrix (at level 1):random part mean fun. for y1
##   matrix[N, n_u_mu] Z_y1_mu_L1;
##   //design matrix (at level 2):fixed part wiv fun. for y1
##   matrix[total_num_clinics, n_a] X_y1_wiv_L2;
##   //design matrix (at level 2):random part wiv fun. for y1
##   matrix[total_num_clinics, n_u_total - n_u_mu] Z_y1_wiv_L2;
##   //design matrix:observed exposures for y2
##   matrix[J, n_g_obs] X_y2_obs;
##   //repeatedly-measured outcome
##   vector[N] y1;
##   //individual-level outcome
##   vector[J] y2;
##   //subject indicator (of N-length)
##   int<lower=1,upper=J> Ind_L1[N];
##   //subject indicator (of total_num_clinics-length)
##   int<lower=1,upper=J> Ind_L2[total_num_clinics];
##   //clinic indicator (of N-length)
##   int<lower=1,upper=total_num_clinics> clinic_L1[N];
##   //location and scale of priors for betas
##   vector[n_b] beta_prior_loc;
##   vector<lower=0>[n_b] beta_prior_scale;
##   //location and scale of priors for alphas
##   vector[n_a] alpha_prior_loc;
##   vector<lower=0>[n_a] alpha_prior_scale;
##   //location and scale of priors for observed exposures for y2
##   vector[n_g_obs] gamma_obs_prior_loc;
##   vector<lower=0>[n_g_obs] gamma_obs_prior_scale;
##   //location and scale of priors for RE exposures for y2
##   vector[n_g_RE] gamma_RE_prior_loc;
##   vector<lower=0>[n_g_RE] gamma_RE_prior_scale;
##   //eta for LKJcorr prior for RE correlations
##   real<lower=0> LKJcorr_prior;
##   //location and scale of priors for RE SDs
##   real sigma_u_prior_loc;
##   real<lower=0> sigma_u_prior_scale;
##   //location and scale of prior for residual SD for y2
##   real sigma_y2_prior_loc;
##   real<lower=0> sigma_y2_prior_scale;
##   //location and scale of prior for residual SD (at level 1) for y1
##   real sigma_epsilon_prior_loc;
##   real<lower=0> sigma_epsilon_prior_scale;
##   }
## parameters {
##   //FE coefficients in mean function for y1
##   vector[n_b] beta;
##   //FE coefficients in within-individual variance function for y1
##   vector[n_a] alpha;
##   //FE coefficients of observed exposures for y2
##   vector[n_g_obs] gamma_obs;
##   //FE coefficients of RE exposures for y2
##   vector[n_g_RE] gamma_RE;
```

```
##   //Cholesky factor of random effect corr matrix
##   //(i.e. corr_u = cholesky_corr_u * cholesky_corr_u')
##   cholesky_factor_corr[n_u_total] cholesky_corr_u;
##   //random effect SDs (lower bound ensures half-Cauchy)
##   vector<lower=0>[n_u_total] sigma_u;
##   //unscaled random effects (N(0,1))
##   matrix[n_u_total, J] z_u;
##   //REs at clinic level
##   vector[total_num_clinics] e;
##   //residual SD for y2 (lower bound ensures half-Cauchy)
##   real<lower = 0> sigma_y2;
##   //residual SD for y1 at level 1 (lower bound ensures half-Cauchy)
##   real<lower = 0> sigma_epsilon;
## }
## transformed parameters {
##   //scaled random effects
##   matrix[J, n_u_total] u;
##   u = (diag_pre_multiply(sigma_u, cholesky_corr_u) * z_u)';
## }
## model {
##   //priors
##   beta ~ normal(beta_prior_loc, beta_prior_scale);
##   alpha ~ normal(alpha_prior_loc, alpha_prior_scale);
##   gamma_obs ~ normal(gamma_obs_prior_loc, gamma_obs_prior_scale);
##   gamma_RE ~ normal(gamma_RE_prior_loc, gamma_RE_prior_scale);
##   //normal() not applicable to matrices, hence to_vector
##   //(treats it like a vector but maintains matrix data type)
##   to_vector(z_u) ~ normal(0, 1);
##   cholesky_corr_u ~ lkj_corr_cholesky(LKJcorr_prior);
##   sigma_u ~ cauchy(sigma_u_prior_loc, sigma_u_prior_scale);
##   sigma_y2 ~ cauchy(sigma_y2_prior_loc, sigma_y2_prior_scale);
##   sigma_epsilon ~ cauchy(sigma_epsilon_prior_loc, sigma_epsilon_prior_scale);
##   //likelihood
##   //NB: if want to model log(sigma) instead, then remove sqrt()
##   //from within-individual variability function
##   y1 ~ normal(X_y1_mu_L1 * beta
##               + rows_dot_product(Z_y1_mu_L1, u[Ind_L1, 1:n_u_mu])
##               + e[clinic_L1],
##               sigma_epsilon);
##   e ~ normal(0, sqrt(exp(X_y1_wiv_L2 * alpha
##              + rows_dot_product(Z_y1_wiv_L2, u[Ind_L2, (n_u_mu + 1):n_u_total]))));
##   y2 ~ normal(X_y2_obs * gamma_obs + u * gamma_RE, sigma_y2);
## }
## generated quantities {
##   corr_matrix[n_u_total] corr_u_complete;
##   vector<lower=-1, upper=1>[n_cov] corr_u;
##   // return correlation matrix (dropping redundant elements)
##   //adapted from brms model code
##   corr_u_complete = multiply_lower_tri_self_transpose(cholesky_corr_u);
##   for (k in 1:n_u_total) {
##     for (j in 1:(k - 1)) {
##       corr_u[choose(k - 1, 2) + j] = corr_u_complete[j, k];
##     }
##   }
```

```
## }
```

Fitting the model

```
params_for_summary <- c("beta",
                        "alpha",
                        "gamma_obs",
                        "gamma_RE",
                        "sigma_y2",
                        "sigma_u",
                        "corr_u",
                        "sigma_epsilon")
chains <- 4

# (include u (etc.) in pars if wish to save e.g. residuals at that level)
stan_fit <- stan(file = "joint_L3.stan",
                 data = data,
                 pars = params_for_summary,
                 chains = chains)
```

Inspecting results

```
print(stan_fit, pars = params_for_summary)

# Launching shinystan to check diagnostics
stan_fit_shiny <- as.shinystan(stan_fit, pars = params_for_summary)

launch_shinystan(stan_fit_shiny)
```

Web Appendix 3

This Web Appendix provides further estimation details for models presented in main manuscript.

Chain lengths and convergence

Each model was fitted using four chains. Convergence to the target distribution was judged via visual diagnostics, the presence (and number and type) of any divergent transitions, and the value of split-$\hat{R}$ (with ≈ 1 suggesting convergence) (12).

We ran each chain for 15,000 iterations, including 5,000 warm-up. The chains were run for such a length that the effective sample size for each parameter of interest indicated a reasonable number of independent draws (≥400) from the posterior distribution. Note that the length of chain is very generous, but was set for the one or two models with relatively high autocorrelation for certain parameters.

Priors

For the fixed effects we used Normal priors, whilst for the SDs for the individual-level random effects, and for the occasion-level SD in models in which this was assumed constant, we used half-Cauchy priors (13, 14), and for the correlation matrices for the random effects at the individual-level we used an LKJcorr prior (4, 15). We used values for these priors designed to be weakly-informative (6): further details follow.

For the fixed effects in the mean function for the repeatedly-measured outcome, and for the mean function for the individual-level outcome, we used the equivalent – had the variables been standardised – of Normal(μ_y, σ = 10) for the intercept and Normal(0, σ = 2.5) for the other predictors, re-scaling as appropriate (9).

Half-Cauchy($x_0 = 0$, $\gamma = 10$) priors were used for the SDs for the individual-level random effects, and for the occasion-level SD in models in which this was assumed constant, whilst a prior of LKJcorr(2) was used for the correlation matrices for the random effects at the individual-level (4, 13-15).

For the fixed effects in the within-individual variability function we used Normal priors. For the intercept, the mean of the prior was the estimated within-individual variance (log-transformed) from a simpler (random slope) model, whilst the priors for the covariates had a mean of zero. For the SD of these priors, we used the equivalent – had the variables been standardised – of $\sigma = 2.25$ for both the intercept and covariates. Since a change of +/- 2SDs (around the mean for the prior of the intercept) would have predicted a within-individual SD of between 0.6 and 51.3 (i.e. 4.6% of distribution of prior would predict values above/below this range), this was judged very weakly informative.

References

1. Chen W, Srinivasan SR, Ruan LT, et al. Adult Hypertension Is Associated With Blood Pressure Variability in Childhood in Blacks and Whites: The Bogalusa Heart Study. American Journal of Hypertension 2011;24(1):77-82.
2. Rosner B, Cook NR, Evans DA, et al. Reproducibility and predictive values of routine blood-pressure measurements in children - comparison with adult values and implications for screening-children for elevated blood-pressure. American Journal of Epidemiology 1987;126(6):1115-1125.
3. UCLA: Statistical Consulting Group. FAQ How do I Interpret a Regression Model When Some Variables are Log Transformed? (https://stats.idre.ucla.edu/other/mult-

pkg/faq/general/faqhow-do-i-interpret-a-regression-model-when-some-variables-are-log-transformed/). (Accessed 6th February 2020).

4. Stan Development Team. Stan User's Guide, version 2.19. 2019.
5. Stan Development Team. Stan reference manual, version 2.19. 2019.(http://mc-stan.org)
6. McElreath R. *Statistical Rethinking: A Bayesian Course with Examples in R and Stan*. Boca Raton, FL: CRC Press; 2016.
7. Betancourt M. Diagnosing biased inference with divergences. 2017;(https://mc-stan.org/users/documentation/case-studies/divergences_and_bias.html)
8. Sorensen T, Hohenstein S, Vasishth S. Bayesian linear mixed models using stan: A tutorial for psychologists, linguists, and cognitive scientists. Quantitative Methods for Psychology. 2016;12(3):175–200.
9. Gabry J, Goodrich B. Prior Distributions for rstanarm Models. 2018. (http://mc-stan.org/rstanarm/articles/priors). (Accessed 7th February 2019 2019).
10. Bürkner P-C. brms: An R package for Bayesian multilevel models using Stan. Journal of Statistical Software. 2017;80(1):1–28.
11. Bürkner P-C. Advanced Bayesian multilevel modeling with the R package brms. The R Journal. 2018;10(1):395–411.
12. Gelman A, Carlin JB, Stern HS, et al. Basics of Markov chain simulation. *Bayesian Data Analysis*. Boca Raton, FL, USA: CRC Press, 2013:275-291.
13. Gelman A. Prior distributions for variance parameters in hierarchical models(Comment on an Article by Browne and Draper). *Bayesian Analysis* 2006;1(3):515-533.
14. Polson NG, Scott JG. On the Half-Cauchy Prior for a Global Scale Parameter. *Bayesian Analysis* 2012;7(4):887-901.

15. Lewandowski D, Kurowicka D, Joe H. Generating random correlation matrices based on vines and extended onion method. *Journal of Multivariate Analysis* 2009;100(9):1989-2001.
